# ^1^H NMR Analysis of Microalgal Long-Chain Alkenones and Development of a Sequential Extraction Protocol for Alkenone Isolation and Purification from *Tisochrysis* Microalgae

**DOI:** 10.1021/acsomega.6c02208

**Published:** 2026-05-13

**Authors:** Sarah M. Maffett, Nazir A. Pamplin, Christian Cornejo, Andre Weaver, Robert K. Nelson, Christopher M. Reddy, Gregory W. O’Neil

**Affiliations:** † Department of Chemistry, 1632 Western Washington University, Bellingham, Washington 98225, United States; ‡ Department of Marine Chemistry and Geochemistry, 10627 Woods Hole Oceanographic Institution, Woods Hole, Massachusetts 02543, United States

## Abstract

Polyunsaturated long-chain alkenones are unique lipids produced by certain species of microalgae with both well-established (i.e., as paleoclimatoligical indicators) and emerging applications (e.g., as a renewable hydrocarbon feedstock). Traditionally, alkenone-based research has relied on gas chromatography (GC) for alkenone detection and quantification, which can require long run times (ca. 1 h), multistep sample preparation, and suffer from coelution. ^1^H NMR was therefore investigated as an alternative method to complement GC for the analysis of alkenones. Using a well-resolved singlet at 2.13 ppm corresponding to the methyl group adjacent to the carbonyl (C=O) on methyl alkenones, alkenones were able to be detected in the various extracts of commercial *Tisochrysis* microalgae, including solutions prepared by soaking the microalgae in CDCl_3_. This allowed for the differentiation between alkenone- and nonalkenone-producing algae by a streamlined CDCl_3_ extraction/^1^H analysis procedure. The ability of different solvents and conditions to extract alkenones from microalgae was also assessed by ^1^H NMR, leading to the development of a sequential extraction protocol as a new method for alkenone isolation and purification.

## Graphical abstract

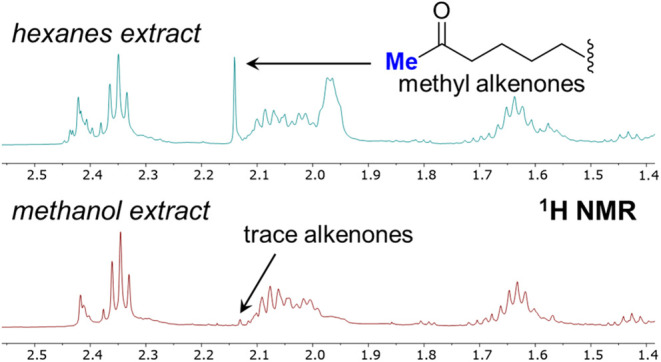

## Introduction

Polyunsaturated long-chain alkenones (alkenones) are a unique class of algae-derived lipids well-known in the field of geochemistry. Their stable structure, characterized by a long hydrocarbon chain (35–42 carbons; typically 37–38 carbons) containing 2–3 *trans* C–C double bonds and terminating in a methyl or ethyl ketone (Figure 1), causes these waxy substances (melting point (mp) ∼ 70 °C) to be retained in the sediment record.^1^ Combined with the sensitivity of alkenone unsaturation to growing temperature, where lower temperatures correspond to greater unsaturation,^2^ alkenones are commonly used as proxies for past sea surface temperatures.^3,4^ Concurrent salinity and partial pressure of CO_2_ can also be estimated by measuring the stable hydrogen and carbon isotope ratios, respectively.^5^ Recently, a combination of alkenones and chlorophyll derivatives were used to reconstruct activities of alkenone-producing algae versus other phytoplankton, and thus changes in salinity and nutrient levels, during the Black Sea Holocene.^6^ Other recent research has revealed distinct alkenone distributions for different phylogenetic groups (e.g., warm-water^7^ versus ice-associated^8^ lineages), providing potentially new opportunities for alkenone-based paleooceanographic investigations.

**Figure 1:**
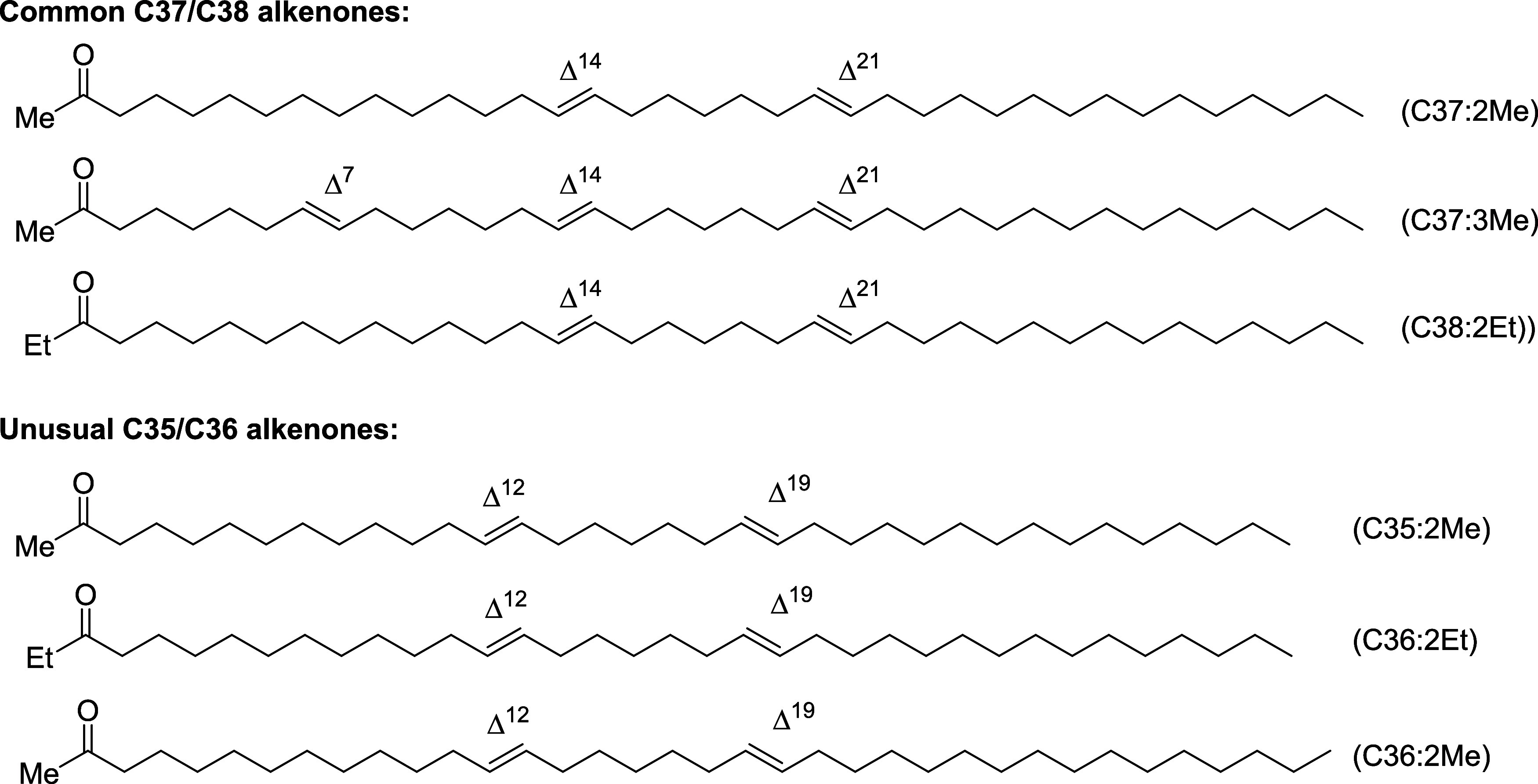
Structures of common C37/C38 and unusual C35/C36 alkenones, where C#:# refers to the length of the carbon chain and number of *trans*-double bonds, Me and Et refer to whether it contains a methyl or ethyl ketone, and Δ^#^ gives the position of the double bond relative to the carbonyl carbon.

Alkenone biosynthesis is limited to certain species of haptophytes, particularly those belonging to the Isochrysidales order, such as *Gephyrocapsa huxleyi*, *Gephyrocapsa oceanica*, *Isochrysis galbana*, *Tisochrysis lutea* (*T-Iso*), and *Ruttnera lamellose*.^9–12^ Among alkenone-producing haptophytes, both *I. galbana* and *T. lutea* are grown industrially at various locations around the world as a primary component of shellfish feed, owing to their favorable growth properties and lipid content.^13^ *T-Iso* has also attracted interest as a source of fucoxanthin,^14^ a carotenoid valued for antioxidant and other biological activities.^15^ Genes associated with fucoxanthin production in *T-Iso* have only recently been elucidated.^16–18^

Motivated by global efforts to identify sustainable hydrocarbon feedstocks, our group began to explore alkenone applications across various industries. Our early efforts focused on renewable fuels.^19–22^ Later, we demonstrated the potential of alkenones as phase-change materials^23^ and as ingredients in personal care products.^24–26^ Part of the reason for shifting from fuel to higher-value, lower-volume products was concerns about cost competitiveness. One way to reduce the cost of alkenones would be to increase the alkenone production in algae. However, alkenone biosynthesis is poorly understood. Only recently has the Suzuki group identified specific enzymes and genes involved in alkenone biosynthesis.^27–30^ During our own work, we have observed significant changes not only in yield but also in the composition of alkenones extracted from *Isochrysis* and *Tisochrysis* algae, even when sourced from the same industrial supplier.^31,32^ These changes are believed to result from differences in cultivation conditions (e.g., nutrient levels, temperature), but we are unaware of any systematic studies examining how these parameters affect alkenone biosynthesis.

To advance alkenone-based technologies and better understand alkenone biosynthesis, reliable methods for quantifying alkenones across different sample types are essential. Traditionally, alkenone levels were measured in the solvent extract of biomass using gas chromatography with flame-ionization detection (GC-FID).^33^ However, the high boiling points of alkenones make GC less than ideal, as it requires high oven temperatures (over 300 °C) and long run times (around 1 h), especially when coelution is a concern.^34^ Other problems, such as irreversible adsorption biases^35^ and sample preparation steps (such as saponification and/or esterification),^33^ have prompted researchers to develop non-GC techniques for alkenone analysis, such as high-performance liquid chromatography-mass spectrometry.^36^

In 2016, Pelusi et al. employed Fourier transform infrared spectroscopy (FTIR) for semiquantitative analysis of total alkenones in algae.^37^ This approach used the signal from the *trans*-C–C double bond to estimate the alkenone content in whole algal cells. Another unique structural feature of alkenones compared to other lipid- and nonlipid algal biomolecules is their ketone functional group. Pelusi and co-workers noted the presence of a signature absorbance at 1705.5 cm^–1^ associated with the C=O stretching vibrational mode in the FTIR spectrum, although the absorbance was low and poorly resolved, making detection and quantification difficult.

Ketones also exhibit characteristic signals in their NMR spectrum. For instance, ketones display peaks around 200 ppm in their ^13^C NMR spectrum corresponding to the carbonyl carbon, away from other lipid molecules like acylglycerols and fatty acids (∼175–180 ppm).^38^ However, the low sensitivity of ^13^C NMR, especially for carbonyl carbons, and challenges with accurately integrating ^13^C NMR spectra make its use for alkenone detection suboptimal. Proton (^1^H) NMR, by contrast, has much higher sensitivity.^39^ We were therefore inspired to investigate how ^1^H NMR could support the ongoing work of our group and others toward the development of various alkenone-based green technologies.

## Results and Discussion

Previously, we have recorded ^1^H NMR spectra of alkenone mixtures isolated from different batches of *Tisochrysis*. Whether consisting of typical C37/C38-^24^ or less common C35/C36 methyl and ethyl alkenones (ref. Figure 1),^31^ the spectra were essentially identical in terms of chemical shifts for the different signals (Figure 2). Each displayed signals between 5.25 and 5.50 ppm corresponding to protons attached to the *trans* carbon–carbon double bonds (C = C). Unfortunately, this is the same region as signals from unsaturated fatty acid *cis*-configured C=C groups, as evidenced by the ^1^H NMR spectrum of an alkenone-free biodiesel (i.e., fatty acid methyl esters; FAMEs) prepared from extracted *T. lutea* acylglycerols (bottom, red trace)^40^ despite their different geometry and location/spacing, making it difficult to distinguish contributions of the different lipid classes to this particular signal. Both alkenone ^1^H NMR spectra show a prominent singlet at 2.1 ppm, also noted by Iglesias et al. in their sophisticated NMR analysis of a *Tisochrysis* lipid mixture as part of a study on antibacterial and antibiofilm activity of microalgal extracts.^41^ Using a suite of NMR experiments (e.g., ^1^H, ^13^C, heteronuclear single quantum coherence (HSQC), and heteronuclear multiple bond correlation (HMBC)) performed on a high-field (600 MHz, 14 T) spectrometer equipped with a cryo-probe, Iglesias and co-workers were able to definitively assign the singlet at 2.13 ppm to the methyl group (Me) adjacent to the ketone of methyl-alkenones. With the benefit of using pure alkenones, other signals in the ^1^H NMR spectra we recorded were readily assigned,^42^ for instance α-keto and allylic methylenes at ∼2.4 and ∼1.9 ppm respectively, with contributions from both methyl- and ethyl alkenones (i.e., H_a–c_ and H_x,y_). For a signal unique to ethyl alkenones, the triplet at 1.05 ppm was assigned to the β-CH_3_ group of ethyl alkenones.

**Figure 2:**
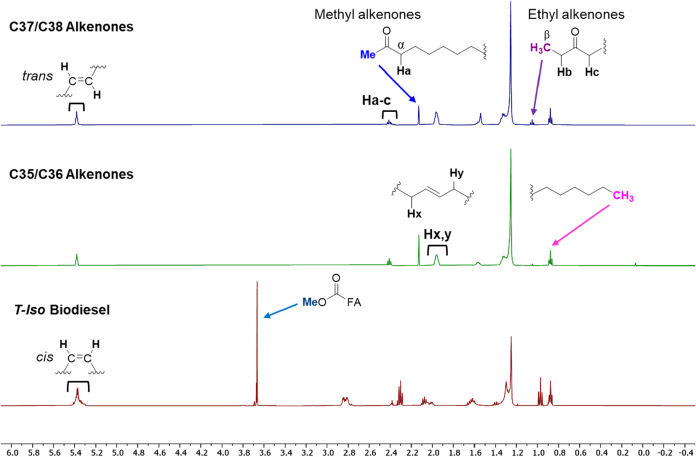
Stacked ^1^H NMR spectra of alkenone mixtures (top and middle) and an alkenone-free biodiesel (i.e., FAMEs) produced from *Tisochrysis* algae (bottom).

Integration values from the ^1^H NMR spectra of pure alkenones we acquired support these signal assignments. For instance, the integration ratio between the signals at ∼5.3 and ∼1.9 ppm is approximately 1:2 (∫ = 1.00:1.81), consistent with hydrogens attached to and adjacent to C=C groups (Figure 3). If the methyl alkenone singlet and ethyl alkenone triplet assignments are correct, both arising from CH_3_ groups, that would indicate a ∼2:1 ratio of methyl/ethyl alkenones in this sample based on integration of those signals (∫ = 0.41:0.22). This number would also be consistent with the integration value obtained for the α-keto hydrogens at ∼2.4 ppm taking into account the ratio of methyl to ethyl alkenones in the sample (2:1) and the different ratio of the CH_3_ to α-keto hydrogens for methyl (3:2) and ethyl (3:4) alkenones (∫ = 0.41 × 2/3 + 0.22 × 4/3 = 0.56).

**Figure 3:**
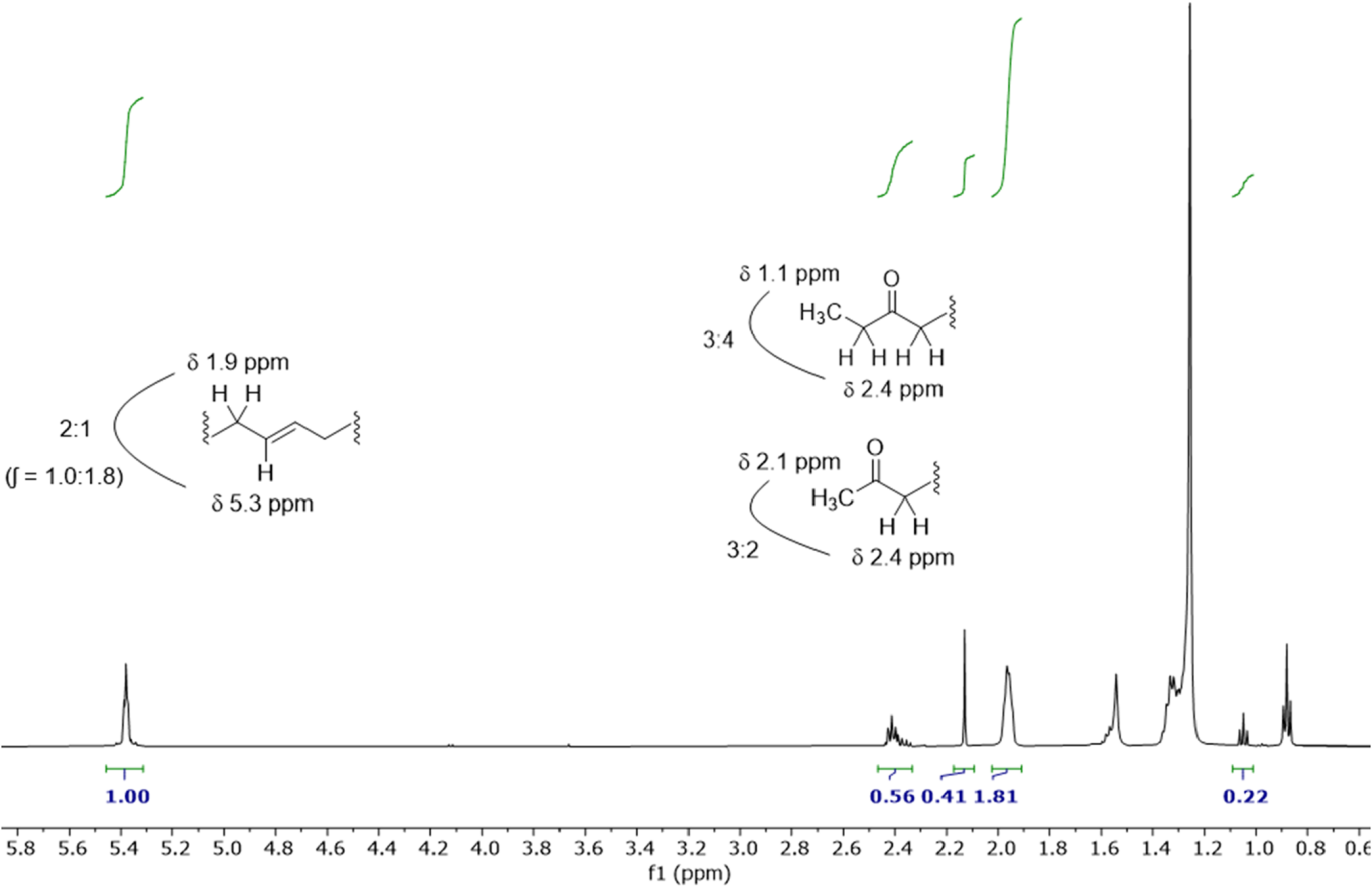
Integrated ^1^H NMR spectrum of pure C37/C38 alkenones. The integration values match those expected based on signal assignments indicated by the partial alkenone structures shown.

As further confirmation of the ^1^H NMR signal assignments, the purified alkenone mixture whose ^1^H NMR spectrum is shown in Figure 3 was analyzed by comprehensive two-dimensional gas chromatography (GC × GC). The exceptional resolution and accuracy of GC × GC serve as a rigorous test for the NMR data. Figure 4 shows the resulting GC × GC chromatogram (plan view). Comparing the areas of the peaks belonging to methyl- (peaks A and B) and ethyl alkenones (peaks E and F) gives a 1.93:1 ratio, very close to that determined by ^1^H NMR (1.86:1). This is important, since the ^1^H NMR spectra were recorded using standard parameters (e.g., 1.0 s relaxation delay) without accounting for differences in relaxation times between signals which can affect integration values. Subsequently, the T1 relaxation times of the signals used to quantify the ratio of methyl to ethyl alkenones was determined to be 3.57 and 2.05 s, respectively from inversion-recovery experiments.^43,44^ The longer relaxation time for the methyl alkenone signal would explain its underintegration relative to the ethyl alkenone signal, causing a slightly lower than actual methyl alkenone/ethyl alkenone ratio. Unless rigorous quantitation is needed, the reduced acquisition time associated with the shorter relaxation delay would generally outweigh the increased time required to ensure accurate integrations.

**Figure 4:**
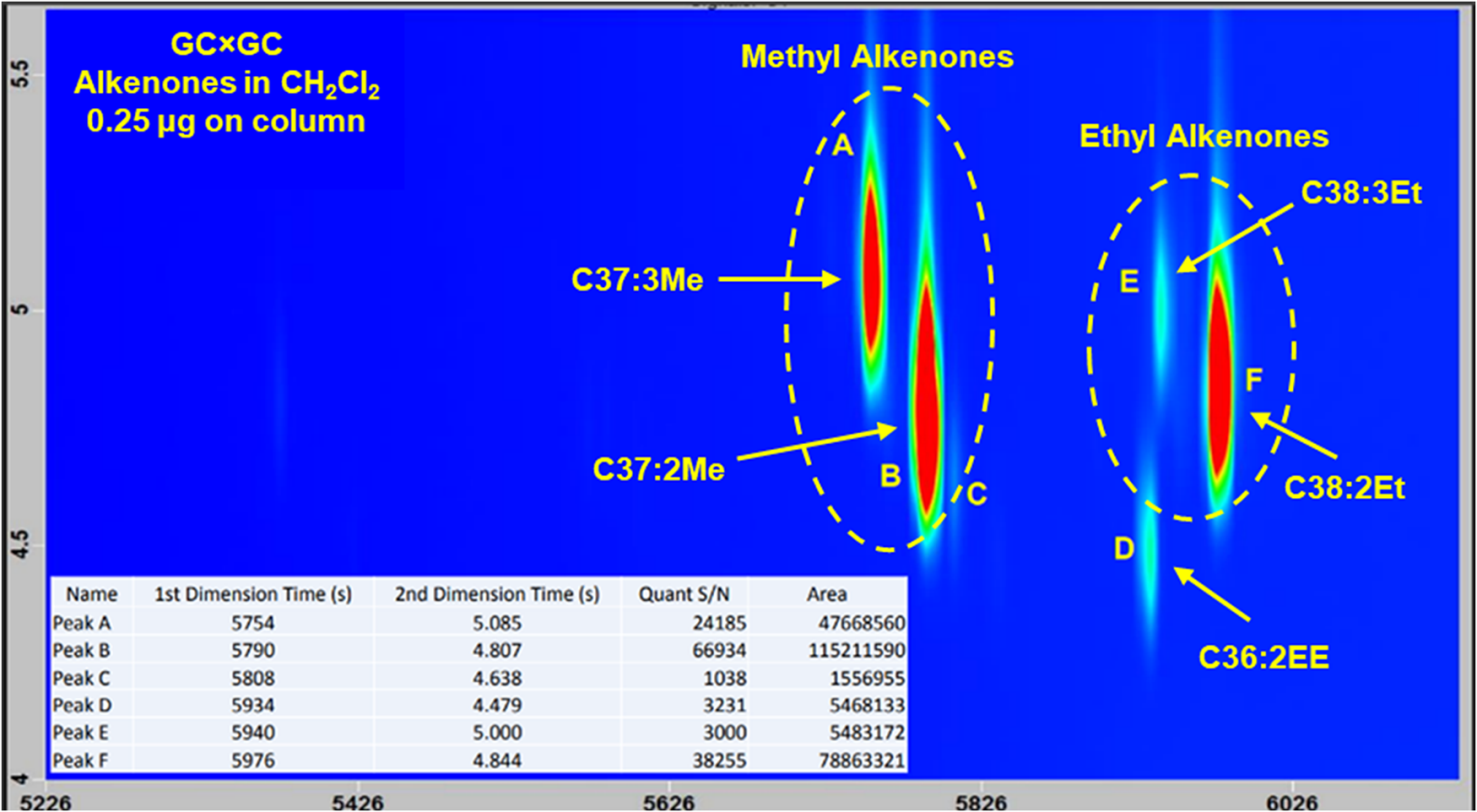
GC × GC chromatogram (plan view) of the purified alkenone mixture whose ^1^H NMR spectrum is shown in Figure 3. The ratio of methyl (peaks A and B) to ethyl alkenones (peaks E and F) by GC × GC was identical to that obtained by integrating the ^1^H NMR signals unique to these compounds (1.9:1). Peak D was assigned to C36:2 ethyl ester (FAEE) based on comparison to reported retention times^45^ and its mass spectrum.^46^

We next investigated whether alkenones could be detected in mixtures such as algal extracts by routine ^1^H NMR analysis on a 500 MHz NMR equipped with a standard broadband probe. For this purpose, *Tisochrysis* microalgae was extracted by Soxhlet with hexanes to produce a “hexanes algal oil” that we have previously described as a mixture of alkenones plus other components like acylglycerols and chlorophylls.^19^ The hexanes algal oil was dissolved in CDCl_3_ at a concentration of 10 mg mL^–1^ and its ^1^H NMR spectrum was recorded (16 scans, 1.5 min acquisition time). Consistent with Iglesias’s data,^41^ the well-resolved singlet at 2.13 ppm from methyl alkenones was easily identifiable within the mixture (Figure 5). Spiking the sample with pure alkenones increased the intensity of this peak, further confirming its assignment.

**Figure 5:**
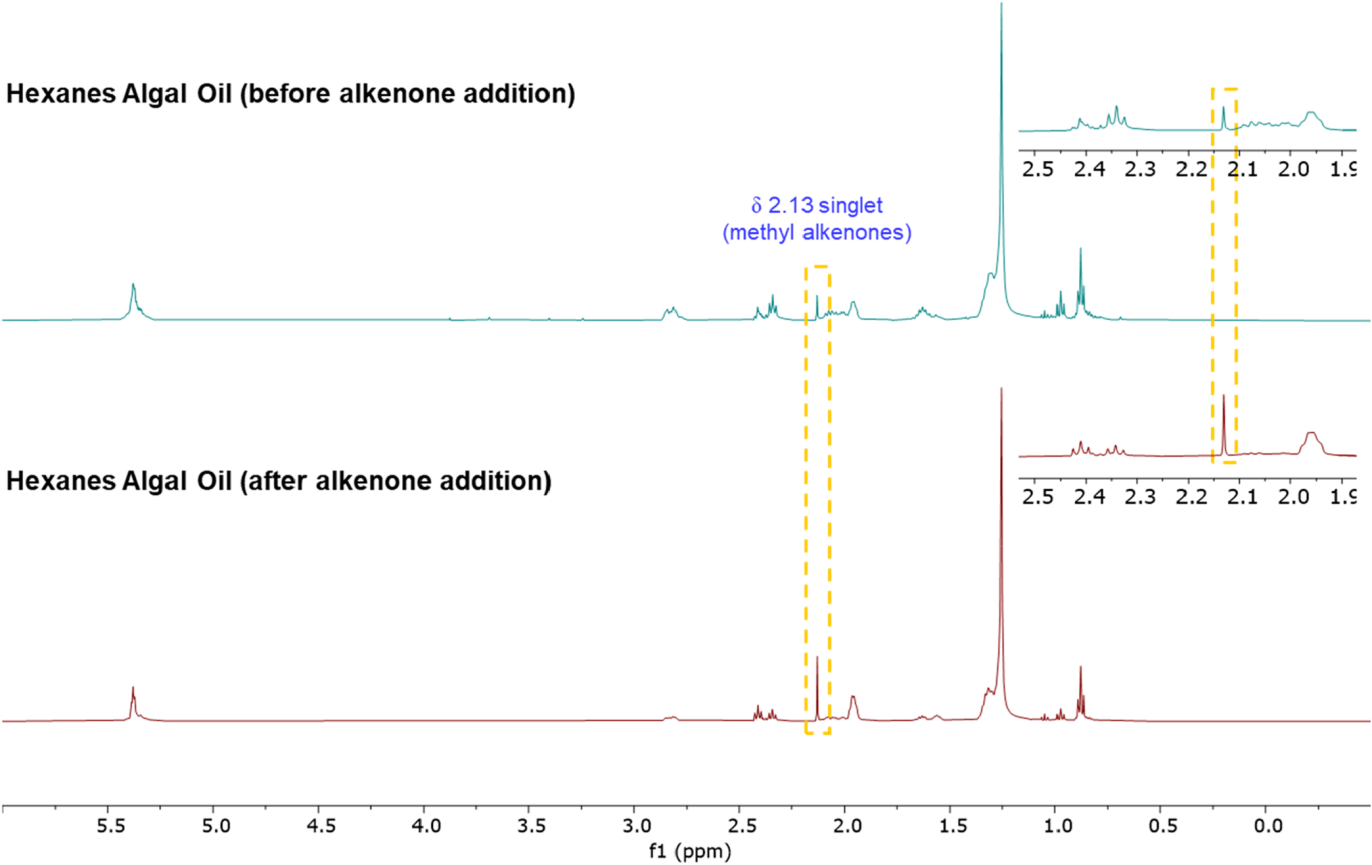
^1^H NMR spectrum of a *Tisochrysis* hexanes extract (hexanes algal oil; top) and that same sample after addition of pure alkenones (bottom). A well-resolved singlet can be see in the hexanes algal oil that we assigned to methyl alkenones, which was confirmed by that signal increasing in intensity upon addition of pure alkenones.

To test the ability of lower-field NMR spectrometers to detect alkenones, ^1^H NMR spectra of *T-Iso* hexanes algal oil as 10 mg mL^–1^ solutions in CDCl_3_ were acquired on both a 7.05 T (300 MHz) and 1.4 T (60 MHz) instrument. As shown in Figure 6, the singlet at 2.13 ppm was clearly visible although not completely resolved in the 300 MHz ^1^H NMR spectrum. The peak was still discernible from the 60 MHz instrument, although the extent of broadening in the 60 MHz spectrum makes its assignment somewhat tenuous. Our recommendation is therefore the use of a ≥300 MHz instrument for detecting alkenones in lipid mixtures by NMR.

**Figure 6:**
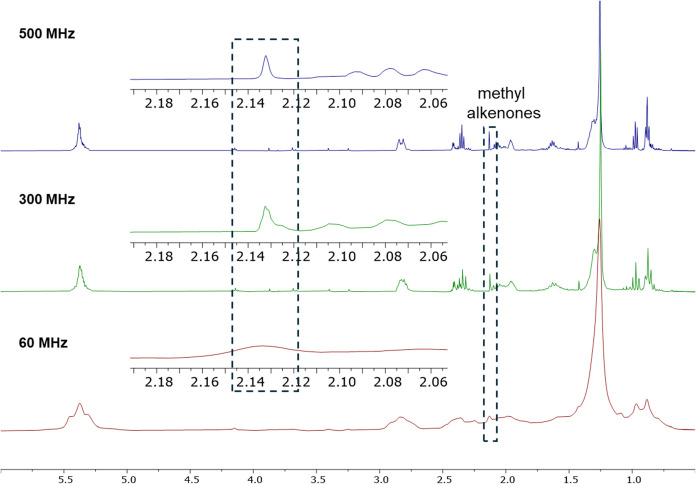
Comparison of NMR field-strength for detecting alkenones within *T-Iso* hexanes algal oil. Spectra were acquired using a 10 mg mL^–1^ solution of hexanes algal oil in CDCl_3_ on an NMR instrument with a proton resonance of 500 MHz (top), 300 MHz (middle), and 60 MHz (bottom).

To simplify the procedure, we wanted to know whether the extraction could be performed in a deuterated solvent, enabling direct NMR analysis of the resulting solution. To that end, 25 mg of dry *Tisochrysis* microalgae was soaked in warm (50 °C) CDCl_3_ (1 mL). The sample was centrifuged to pellet the algae and the solution was then transferred to an NMR tube for analysis. Indeed, alkenones were detectable, with the ^1^H NMR spectrum obtained resembling that from the two-solvent extraction/analysis (Figure 7). For comparison, a nonalkenone-producing algae widely used for research purposes,^47^ *Nannochloropsis*, was extracted and analyzed in the same way. The resulting ^1^H NMR spectra contained no singlet at 2.1 ppm, demonstrating the ability of ^1^H NMR to distinguish between alkenone and nonalkenone-producing algae.

**Figure 7:**
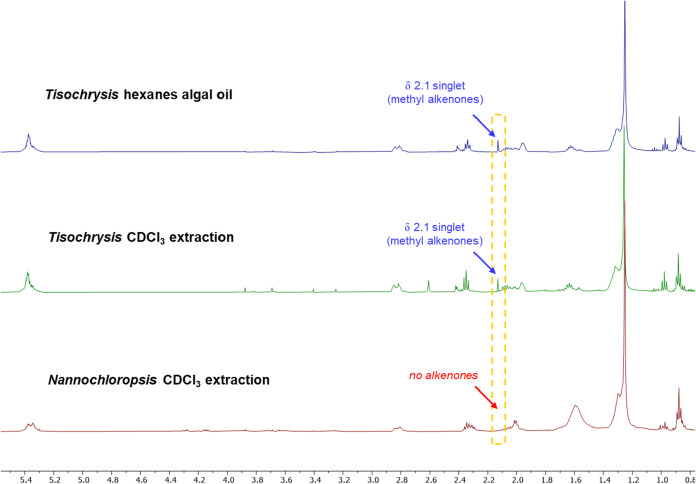
^1^H NMR spectra of *Tisochrysis* hexanes algal oil dissolved in CDCl_3_ (top), *Tisochrysis* CDCl_3_ extraction solution (middle), and *Nannochloropsis* CDCl_3_ extraction solution (bottom). The *Nannochloropsis* lacked a distinctive singlet at 2.13 ppm since this algae does not produce alkenones.

As a further demonstration of the utility of this ^1^H NMR-based method for alkenone detection, we investigated its use to support the development of an improved method for the isolation of alkenones from microalgal biomass. Previously, following Soxhlet extraction with hexanes, a sequence of saponification (KOH, 50% w/w, 3 h), chromatography on silica, decolorization using montmorillonite clay, and crystallization was used to isolate alkenones from microalgal biomass.^21,23,40^ While this process is somewhat lengthy, it has provided sufficient amounts of pure alkenones to be used in various demonstration studies. An ideal process would avoid the use of corrosive KOH and the waste-generating chromatography/decolorization steps.

To achieve this goal, a sequential extraction protocol was designed to produce a material enriched with alkenones, making it easier to isolate. Other researchers have reported sequential algae extractions to selectively generate multiple product streams containing specific components.^48,49^ Yet, alkenones were not included in that previous work despite using algae that produce alkenones in those studies.^50^ We were therefore motivated to develop a sequential extraction protocol for isolating alkenones from algal biomass using ^1^H NMR to monitor the alkenones throughout the process.

We anticipated that alkenones would be insoluble in a polar solvent such as methanol, allowing other components to be extracted and separated from the alkenones retained in algal biomass. Commercially available dry *Tisochrysis* biomass was sequentially extracted by Soxhlet using methanol, followed by hexanes. While the hexane extract was clearly more enriched in alkenones, indicated by the prominent singlet at 2.1 ppm in its ^1^H NMR spectrum (ref. Figure 2), somewhat surprisingly, alkenones were also detected in the methanol extract (Figure 8).

**Figure 8:**
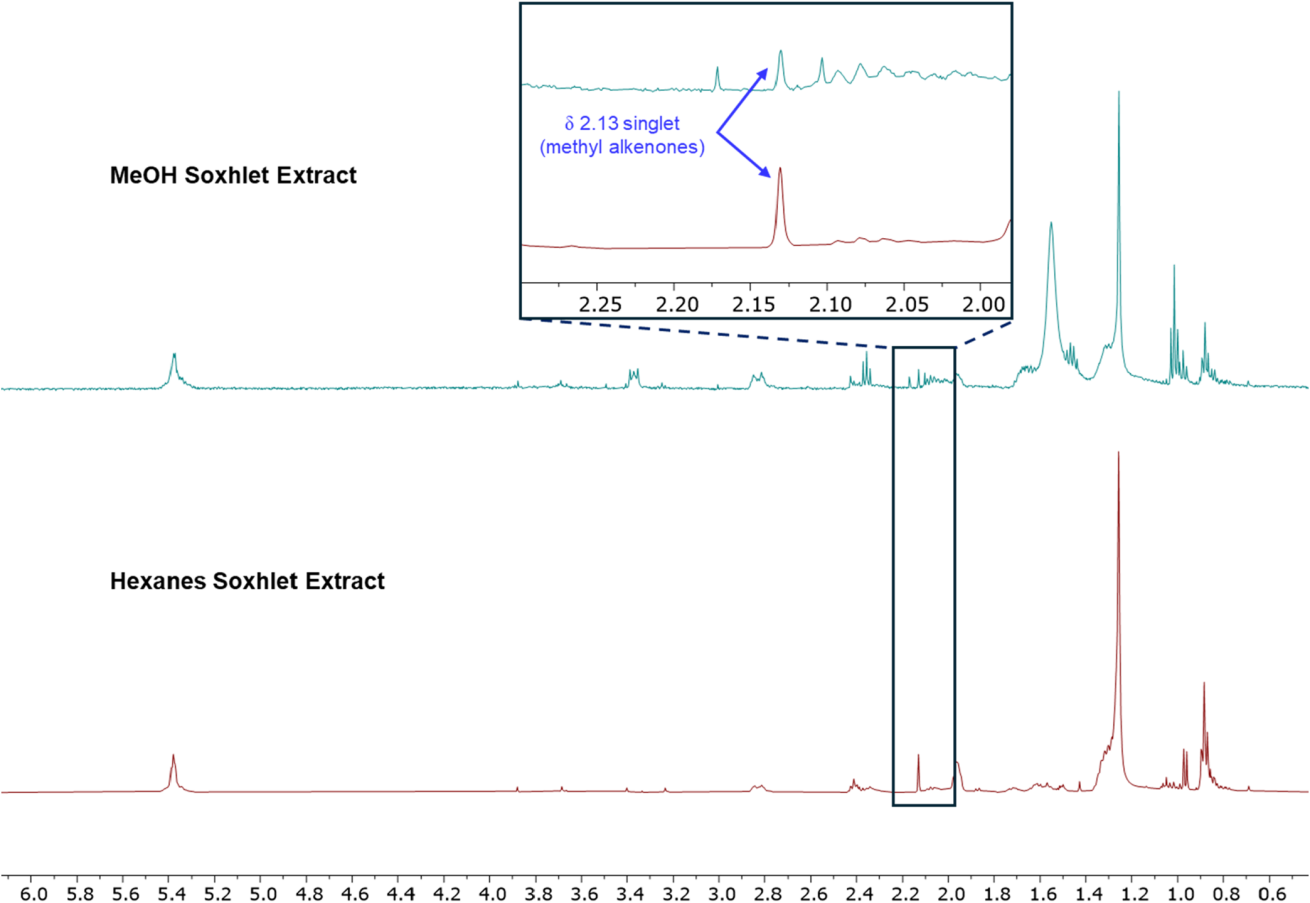
^1^H NMR spectra of extracts obtained by Soxhlet extraction using first methanol (top, blue trace) followed by hexanes (bottom, red trace). Both spectra showed evidence of alkenones, although the hexanes extract appeared more enriched in alkenones based on the relative intensity of the singlet at 2.1 ppm.

Since solubility increases with temperature, we wondered whether performing the initial methanol extraction at room temperature (rt) rather than by Soxhlet extraction would prevent alkenones from being extracted. To test this, samples of *Tisochrysis* were extracted with methanol as well as ethanol and isopropanol by simply soaking the algae biomass in these solvents at room temperature (rt). After the removal of the algae and solvent, the extracts were dissolved in CDCl_3_ and analyzed by ^1^H NMR (Figure 9). According to the results, reducing the extraction temperature increased selectivity, with rt methanol (and ethanol) extracting only a small, but detectable, amount of alkenones. Extraction with isopropanol at rt, however, resulted in fewer alkenones being retained in the algal biomass, presumably due to its lower polarity and thus higher alkenone solubility.

**Figure 9:**
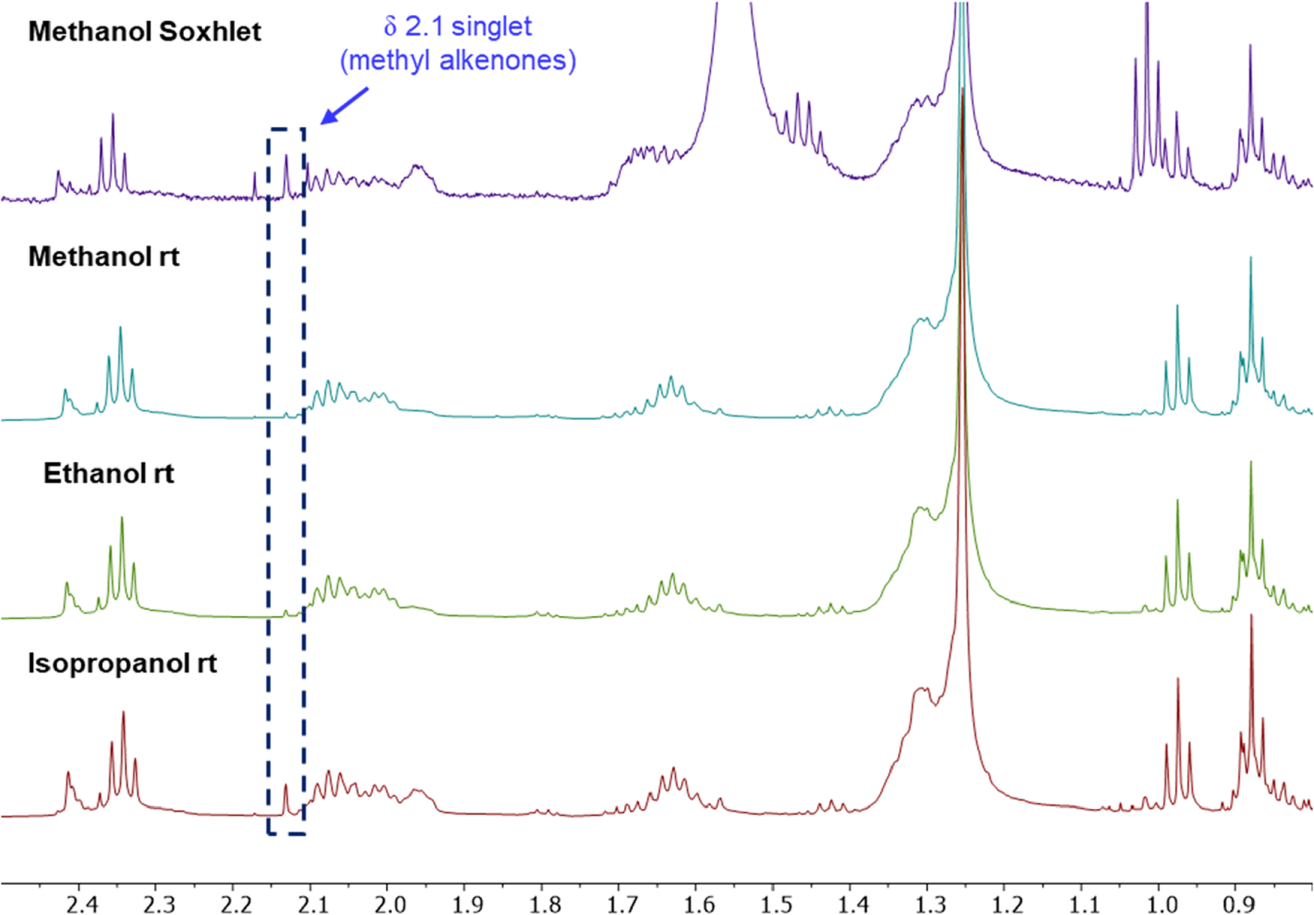
^1^H NMR comparison of *Tisochrysis* extracts performed using different alcohol solvents and via Soxhlet or at room temperature. Both the methanol Soxhlet (top) and isopropanol room temperature (rt) extracts had noticeable quantities of alkenones, indicated by the singlet at 2.1 ppm. This singlet was barely present in the methanol and ethanol rt extracts indicating a very small amount of alkenones were extracted with these solvents. The *y*-axis for each spectrum was scaled so that the intensity of nonalkenone signals were similar (e.g., triplets at 2.35 and 0.98 ppm, multiplets at 2.08 and 1.63 ppm).

For a comparison to more traditional analyses, the solutions used to acquire the ^1^H NMR spectra in Figure 9 were analyzed by GC-FID after transesterification (MeOH, H_2_SO_4_). Consistent with the ^1^H NMR data, signals corresponding to alkenones were detected in the isopropyl alcohol extract (Figure 10). Interestingly, no alkenones were detected in the chromatograms of the rt methanol and ethanol extracts, whereas ^1^H NMR suggested that a small amount was present. Nonetheless, both data sets indicate that an initial rt methanol or ethanol extraction would be effective for separating alkenones from other biomolecules in the algae, for instance, fatty acid components, as evidenced by the fatty acid methyl ester (FAME) signals in the GC chromatograms.

**Figure 10:**
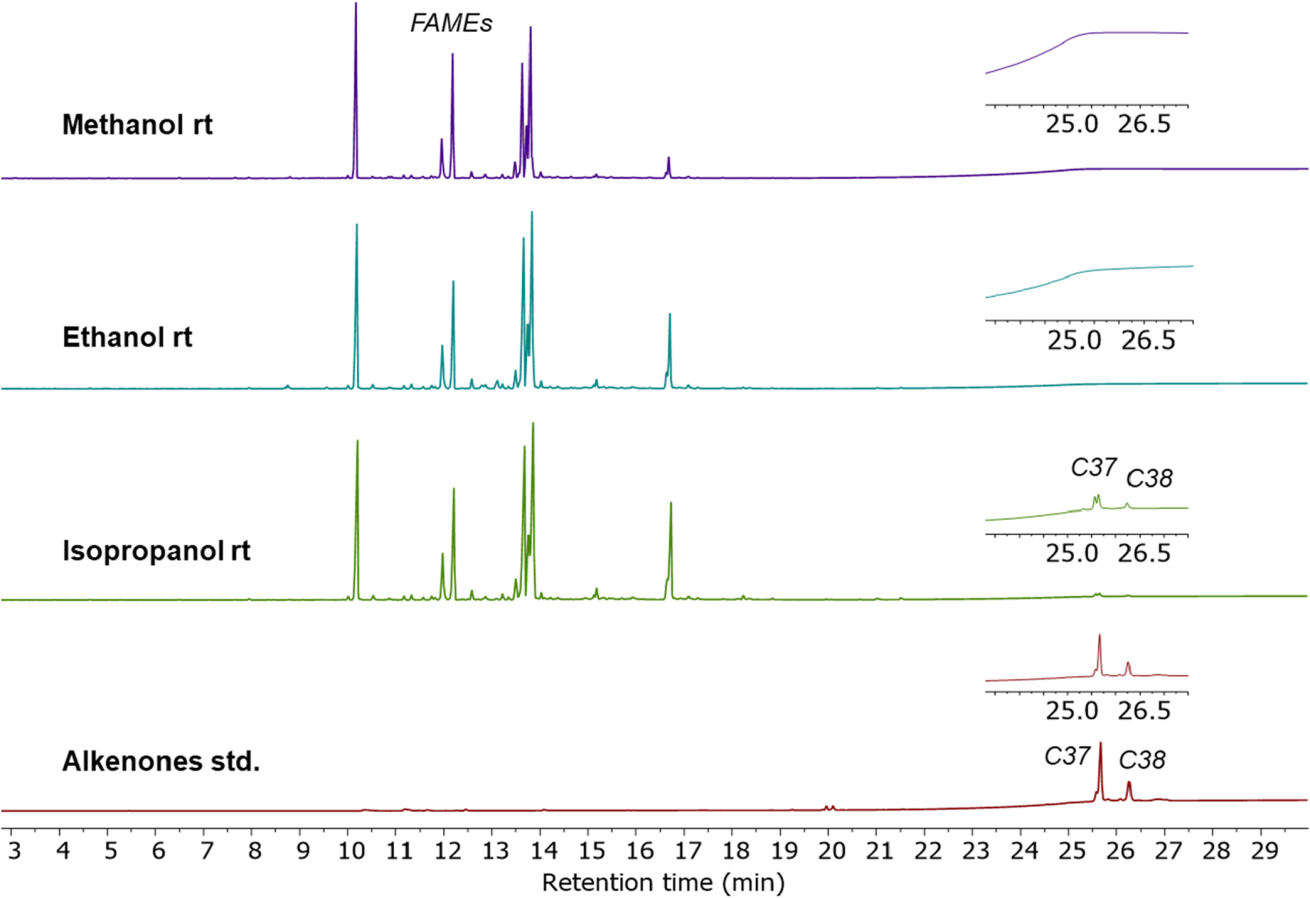
GC-FID chromatograms of materials extracted with different alcohol solvents at room temperature (rt) following transesterification along with an alkenone standard (bottom chromatogram). Only the isopropanol extract showed signals corresponding to alkenones (C37 and C38 based on retention times), whereas all three contained fatty acid components, detected after their conversion to fatty acid methyl esters (FAMEs).

As an initial test for using a sequential extraction technique to streamline alkenone isolation from algae, a cellulose thimble was packed with 10 g of *Tisochrysis* and submerged in methanol for 24 h. The resulting dark-green solution was decanted, and the process was repeated once more. The thimble was then transferred to a Soxhlet extraction apparatus and extracted with hexane for 24 h. Removal of the hexanes on a rotary evaporator then produced a black/brown solid in 5.2% yield (w/DW algae) from the starting algae. Before analysis by NMR, the sample was spiked with a known amount of 1,3,5-trimethoxybenzene (TMB) as an internal standard. TMB was chosen because it was anticipated that the signal corresponding to the aromatic hydrogen (H_A_ in Figure 11) would be well-resolved. Additionally, since this signal corresponds to 3 equiv hydrogens, just as the identifiable alkenone CH_3_ groups, integration values can be directly compared. In this way, using the integrations for the signals corresponding to TMB^51^ and methyl- and ethyl alkenones, the total alkenone content of the solid was calculated to be 68% w/w. The yield of alkenones extracted from the algae would then be 3.5% w/DW, which is on the higher end of what we have reported previously (1.8–3.5%).^18,20,22,30,31,39,52^

**Figure 11:**
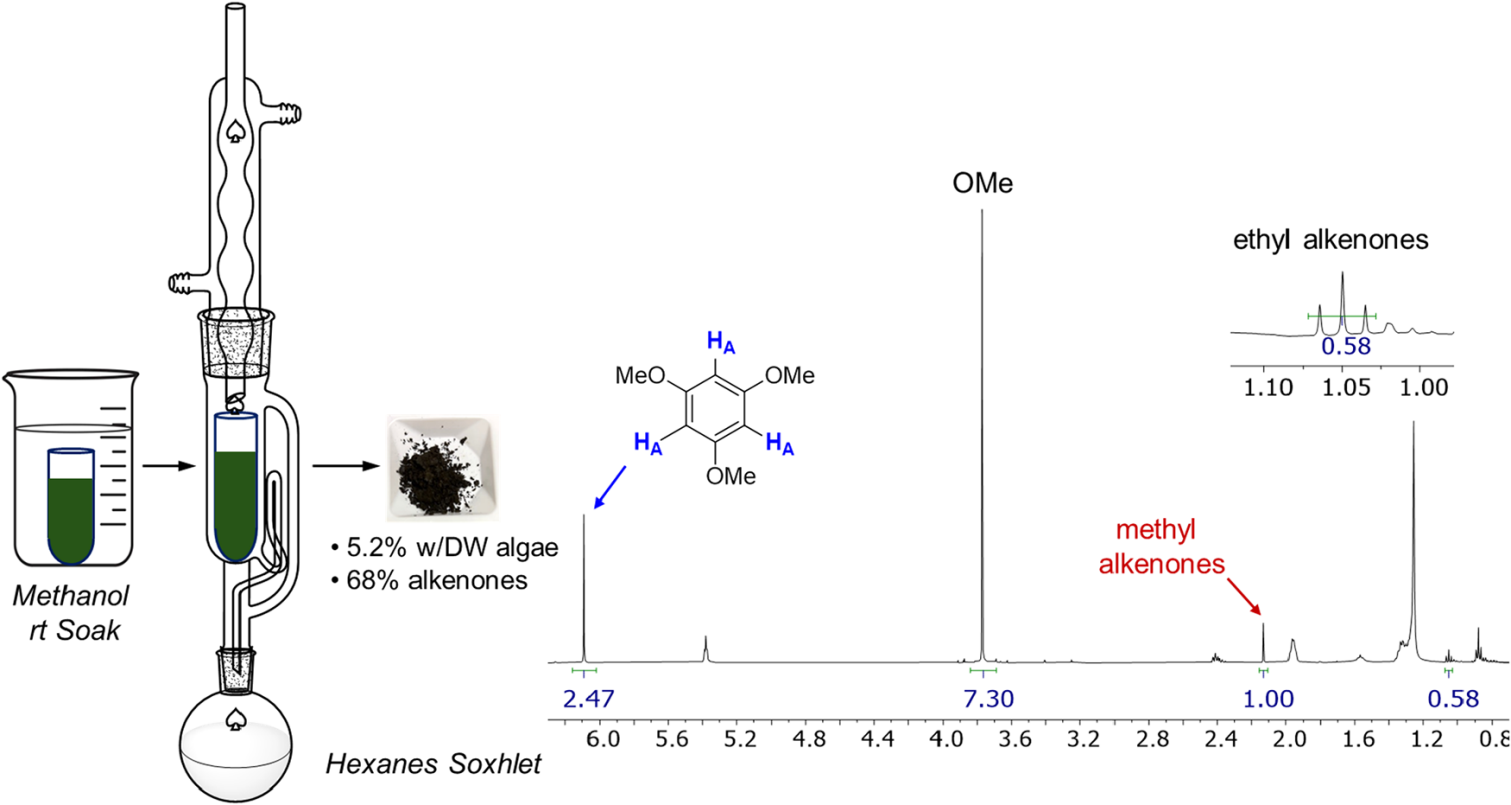
Sequential extraction of *Tisochrysis*. The dry algae were loaded into a cellulose thimble and soaked in methanol at room temperature before being extracted with hexane via Soxhlet. ^1^H NMR analysis of the resulting black solid indicated a total alkenone content of 68% w/w based on integration values assigned to methyl- and ethyl alkenones along with 1,3,5-trimethoxybenzene that was added as an internal standard.

We were concerned that overlapping signals from nonalkenone components in the 1.05 region could have inflated the integration value assigned to ethyl alkenones (ref Figure 11, NMR expansion). For this reason, the solid material we obtained from the MeOH/hexanes sequential extraction was analyzed by GC-FID as an alternative method for quantifying the methyl-to-ethyl alkenone ratio. On the basis of the results from GC-FID, the ratio was 2.8:1 (Me/Et), which is significantly higher than what was obtained by NMR (1.7:1) and would be consistent with overintegration of the ethyl alkenone signal. This may therefore represent a limitation of NMR for quantifying total alkenone amounts in mixtures, however, potentially surmountable by further data processing (e.g., deconvolution techniques, baseline correction, etc.). Using the Me/Et alkenone ratio from GC-FID, the alkenone content of the solid was calculated to be 3.0%, which is closer to the average value we have reported previously (2.8%). Compared to simply extracting with hexanes, which produced an algal oil that was 15–20% w/w alkenones, the data indicate that sequential extraction with methanol followed by hexanes is able to produce a more alkenone-enriched extract with complete recovery of alkenones from the algal biomass. Efforts are ongoing to further investigate this process as part of a biorefinery approach toward valorizing alkenones as a renewable and sustainable hydrocarbon feedstock.

In conclusion, ^1^H NMR offers a convenient method for detecting alkenones in microalgal extracts. Compared to GC, which has traditionally been used for this purpose, one benefit of using NMR is the avoidance of derivatization reactions for acylglycerol components, which generally involve highly acidic or basic reagents and generate waste. Acquisition of ^1^H NMR spectra can also be faster than GC run times for alkenone mixtures because of their high molecular weights, particularly when milligram quantities of sample are available. Even with lesser sample amounts, ^1^H NMR might still be employed by extending acquisition times or incorporating other techniques for signal enhancement.^53–57^ Challenges with ^1^H NMR for alkenone analysis include distinguishing signals for individual alkenones within the mixture such as methyl- versus ethyl alkenones and di- versus triunsaturated. Therefore, ^1^H NMR might be best suited to investigations like those presented here such as differentiating between alkenone- and nonalkenone species of algae and tracking bulk alkenones through various processes. However, more sophisticated NMR experiments may be capable of extending NMR to beyond these applications.

## Methods

### General

NMR: Spectra were recorded on a Bruker 500 MHz spectrometer in CDCl_3_ as solvent; chemical shifts are given in ppm; residual CHCl_3_ was used as a reference (7.26 ppm). GC-FID: Agilent 7890. GC × GC-TOF: Leco Pegasus 4D equipped with a Hewlett-Packard 6890 GC (TOFMS) and 7890 GC (FID system). Reagents and solvents used were purchased from Fisher Scientific and used as received. If washing any glassware, including NMR tubes, with acetone, it is recommended to thoroughly dry the glassware in an oven (∼80 °C) before using to avoid any signal overlap between acetone (δ 2.17 ppm) and alkenones (δ 2.13 ppm) in ^1^H NMR spectra.^58^

### Microalgae

*Tisochrysis* (sold as *IsoPrime*; Batch No. 0301-TISO108) was purchased from Proviron (Hemiksem, Belgium). *Nannochloropsis* was supplied by Necton S.A. (Olhão, Portugal; Lot No. L3250122). The algae were received as a dry-milled powder that were green in color.

### Soxhlet Extraction

Approximately 10 g of *Tisochrysis* was loaded into a cellulose thimble (single thickness, 43 × 123 mm^2^) and extracted with either hexanes or methanol (∼200 mL) by Soxhlet. The Soxhlet was allowed to cycle until the color of the solvent became light yellow (24–48 h). Removal of the solvent on a rotary evaporator produced a dark green near-black material referred to as algal oil.

### Room Temperature Algae Extraction

For extractions performed with hexanes or methanol, approximately 2 g of *Tisochrysis* was added to a centrifuge tube followed by solvent (∼20 mL) and the mixture was agitated using a stir bar and stirring plate for 24 h. The tube was then centrifuged (5000 rpm for 5 min) before decanting the solution and concentrating on a rotary evaporator. For extractions using CDCl_3_, 25 mg of *Tisochrysis* was placed in a centrifuge tube followed by CDCl_3_ (1 mL) and the tube was warmed to 50 °C for 6 h. The tube was then centrifuged (5000 rpm for 5 min) before transferring the supernatant into a standard NMR tube (8″ L, 5 mm OD).

### NMR Analysis

^1^H NMR spectra of purified alkenones and extracts were obtained on a Bruker 500 MHz instrument under ambient conditions. With the exception of the direct CDCl_3_ algae extracts, solutions were prepared by dissolving samples (∼10 mg) in CDCl_3_ (0.7 mL), which also served as internal reference (shift value of residual CHCl_3_ at 7.26 ppm). Spectra were then recorded using a 30° pulse angle (P1), 16 scans, and 1.0 s relaxation delay (D1). For quantitative NMR analysis using TMB as an internal reference, D1 was increased to 18 s by inputting this new value into the acquisition parameters prior to acquisition to account for T1 relaxation times of integrated signals (6.1 ppm = 3.52 s, 2.1 ppm = 3.57 s, 1.1 ppm = 2.05 s; ref Figure 10) determined from inversion-recovery experiments.^46^

### Analysis by One-Dimensional Gas Chromatography with Flame-Ionization Detection (GC-FID)

Purified alkenones and extracts were analyzed using GC-FID. Prior to analysis by GC, extracts were transesterified according to the method of Antolín.^59^ Briefly, approximately 10 mg of the extract was dissolved in CHCl_3_ (1 mL) to which was added a solution of H_2_SO_4_ in MeOH (2% v/v, 1 mL). The mixture was then heated to 80 °C and stirred for 1 h. After cooling to room temperature, water (1–2 mL) was added, the mixture was gently shaken, and the two phases were separated. The organic phase was then transferred to an autosampler vial. Pure alkenone solutions were prepared by dissolving 1–2 mg in 1–2 mL CHCl_3_. Samples of solutions (1 μL) were injected cool-on-column and separated on a 100% dimethyl polysiloxane capillary column (Agilent HP-5, 30 m length, 0.32 mm ID, 0.25 μm film thickness) with He as the carrier gas at a constant flow of 5.0 mL min^–1^. The GC oven was programmed from 70 °C and ramped at 10 °C min^–1^ to 320 °C (5 min hold).

### Analysis by Comprehensive Two-Dimensional Gas Chromatography and High-Resolution Time-of-Flight Mass Spectrometer (GC × GC-TOF HRMS)

Two Leco Pegasus 4D GC × GC systems were used in this study coupled with a TOFMS and a FID, respectively. They were equipped with a Hewlett-Packard 6890 GC (TOFMS) and a 7890 GC (FID system) and configured with split/splitless autoinjectors (7683B series) and a dual-stage cryogenic modulator (Leco, Saint Joseph, Michigan). Samples were injected in splitless mode. The modulator operates with a cold and a hot jet. The cold jet gas was dry N_2_, chilled with liquid N_2_. The hot jet was operated with air that was heated at 5 °C above the temperature of the main GC oven. Two capillary GC columns were fitted in each GC × GC instrument. The first-dimension column was a nonpolar Restek Rxi-1 ms (60 m length, 0.25 mm ID, 0.25 μm film thickness), and the second-dimension separations were performed on a 50% phenyl polysilphenylene-siloxane column (SGE BPX50, 1.0 m length, 0.10 mm ID, 0.1 μm film thickness).

For GC × GC-TOF analysis, the temperature program of the main oven started isothermal at 45 °C (10 min) and was then ramped from 100 to 340 °C at 1.50 °C min^–1^. The hot jet pulsed width was 1.0 and the modulation period was 6.0 s with a 2.00 s cooling period between stages. The second-dimension oven was programmed from 105 °C (10 min) to 345 °C at 1.50 °C min^–1^. The TOFMS data were sampled at an acquisition rate of 100 spectra per second. The transfer line from the second oven to the TOFMS was deactivated fused silica (0.5 m length, 0.18 mm ID), constantly held at 315 °C. The TOF detector voltage was 1335 V and the source temperature 220 °C. The mass spectrometer employs 70 eV electron ionization and operates at a push pulse rate of 5 kHz allowing sufficient signal averaging time to ensure good signal-to-noise ratios while still operating at a high enough data acquisition rate to accurately process (signal average) spectra from the peaks eluting from the second-dimension column in this high-resolution separation technique with second dimension peak widths on the order of 50–200 ms.

### Sequential *Tisochrysis* Extraction and Alkenone Quantification

*Tisochrysis* (9.97 g) was added to a cellulose thimble (single thickness, 43 × 123 mm^2^) followed by a small amount of cotton to seal the algae within the thimble. The thimble was then placed in a beaker (500 mL) and submerged in methanol (200 mL) for 24 h. The resulting green methanolic solution was decanted and the process was repeated. After decanting a second time, the thimble was placed in a Soxhlet extraction apparatus and extracted with hexanes, allowing the Soxhlet to cycle for 24 h. Removal of the hexanes produced a black solid (0.52 g) that was then analyzed by GC-FID and ^1^H NMR. The ^1^H NMR sample consisted of the black solid (16 mg) along with 1,3,5-trimethoxybenzene (5 mg, 0.03 mmol) in 0.7 mL CDCl_3_. According to ^1^H NMR, the molar ratio of 1,3,5-trimethoxybenzene to methyl alkenones was 2.5:1. Using an approximate molar mass of 530 g mol^–1^, this corresponds to 6.9 mg of methyl alkenones in the sample. From GC-FID, the ratio of methyl- to ethyl alkenones was 2.8:1, giving 2.5 mg of ethyl alkenones (MW ∼ 544 g mol^–1^) present and 9.4 mg total alkenones (59% w/w).

## Supplementary Material



## Notes

Copies of NMR spectra, data from inversion-recovery experiments for T1 determination, and mass spectra (PDF)
