# Correction to “^1^H NMR Analysis of Microalgal Long-Chain Alkenones and Development of a Sequential Extraction Protocol for Alkenone Isolation and Purification from *Tisochrysis* Microalgae”

**DOI:** 10.1021/acsomega.6c07932

**Published:** 2026-07-22

**Authors:** Sarah M. Maffett, Nazir A. Pamplin, Christian Cornejo, Andre Weaver, Robert K. Nelson, Christopher M. Reddy, Gregory W. O’Neil

The following needs to be added to the Acknowledgment:

C.C. and A.W. were supported by NSF REU site award CHE-2243968.

