# Supplementary material for: 1H NMR Analysis of Microalgal Long-Chain Alkenones and Development of a Sequential Extraction Protocol for Alkenone Isolation and Purification from Tisochrysis Microalgae
Source: ACS Omega. 2026 May 13;11(20):30087–99. doi: 10.1021/acsomega.6c02208 (PMC13216939; doi:10.1021/acsomega.6c02208)
Supplement: Supplementary file 1 [file ao6c02208_si_001.pdf]

Supporting Information for: ^{1}H NMR Analysis of Microalgal Long-Chain Alkenones and Development of a Sequential Extraction Protocol for Alkenone Isolation and Purification from *Tisochrysis* Microalgae

Sarah M. Maffett,[a] Nazir A. Pamplin,[a] Christian Cornejo,[a] Andre Weaver,[a] Robert K. Nelson,[b] Christopher M. Reddy,[b] and Gregory W. O'Neil,*,[a]

[a] *Department of Chemistry, Western Washington University, Bellingham, WA, 98225 (USA)*

[b] *Department of Marine Chemistry and Geochemistry, Woods Hole Oceanographic Institution, Woods Hole, MA, 02543 (USA)*

*Email: *oneilg@wwu.edu*

Contents:

Item	Page
Alkenones ^{1}H NMR Spectrum	S1
Alkenones COSY Spectrum	S2
Alkenones ^{13}C NMR Spectrum	S2
Alkenones HMBC	S3
Alkenones HSQC	S3
Alkenones ^{1}H T1 Relaxation Time Determination	S4-5
Mass Spectra for Alkenones and Alkenoate from GC×GC Analysis	S6-8

Figure S1. NMR spectra of alkenones.

^{1}H NMR spectrum ($CDCl_3$, 500 MHz)

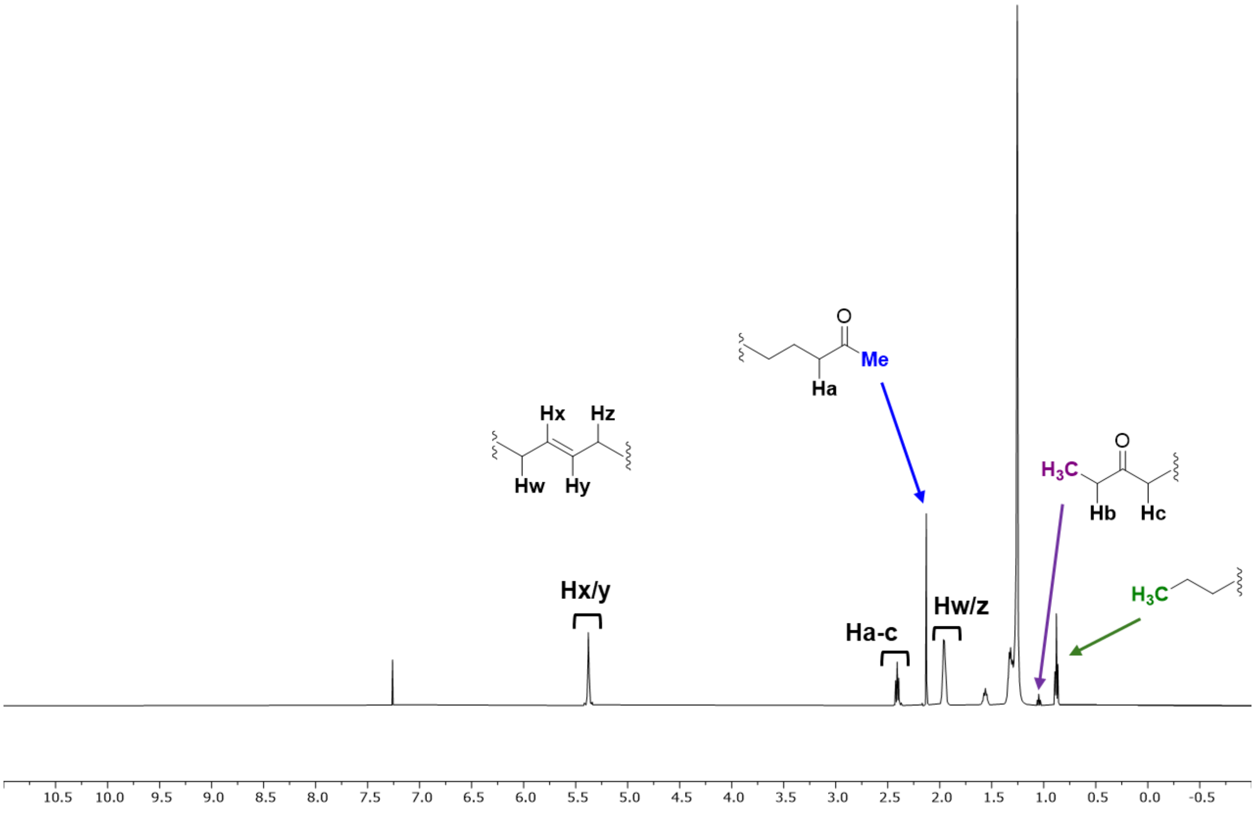

COSY spectrum (CDCl$_3$, 500 MHz)

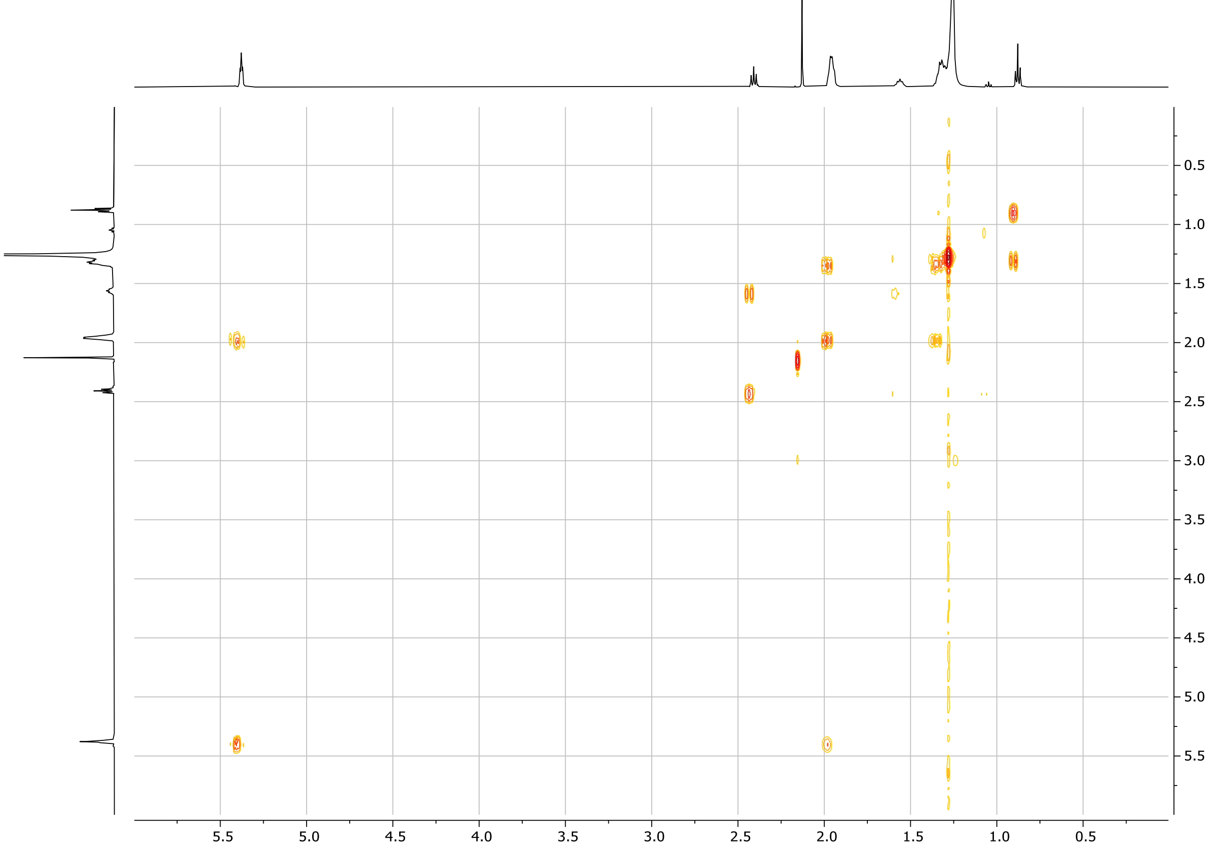

^{13}C NMR spectrum (CDCl$_3$, 126 MHz)

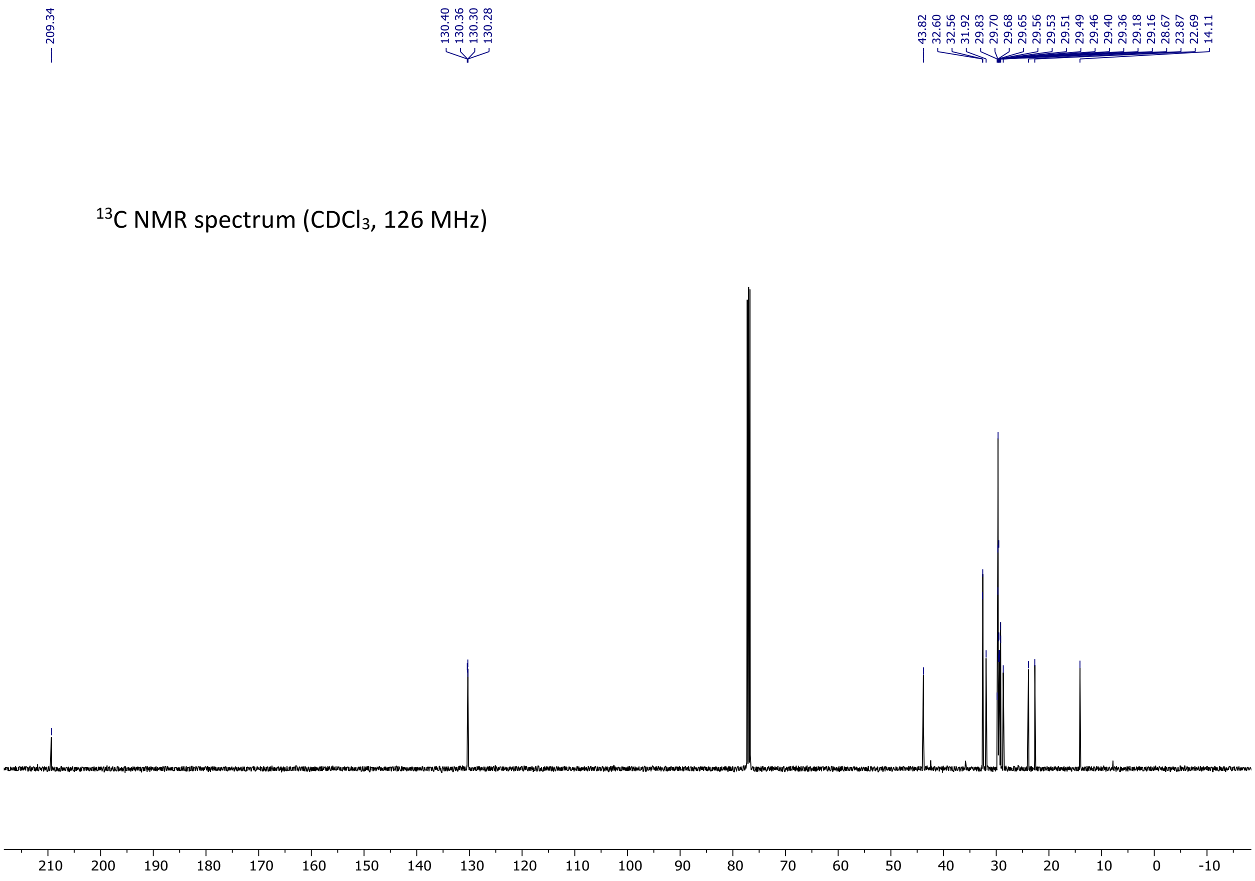

HMBC spectrum ($CDCl_3$, 500 MHz)

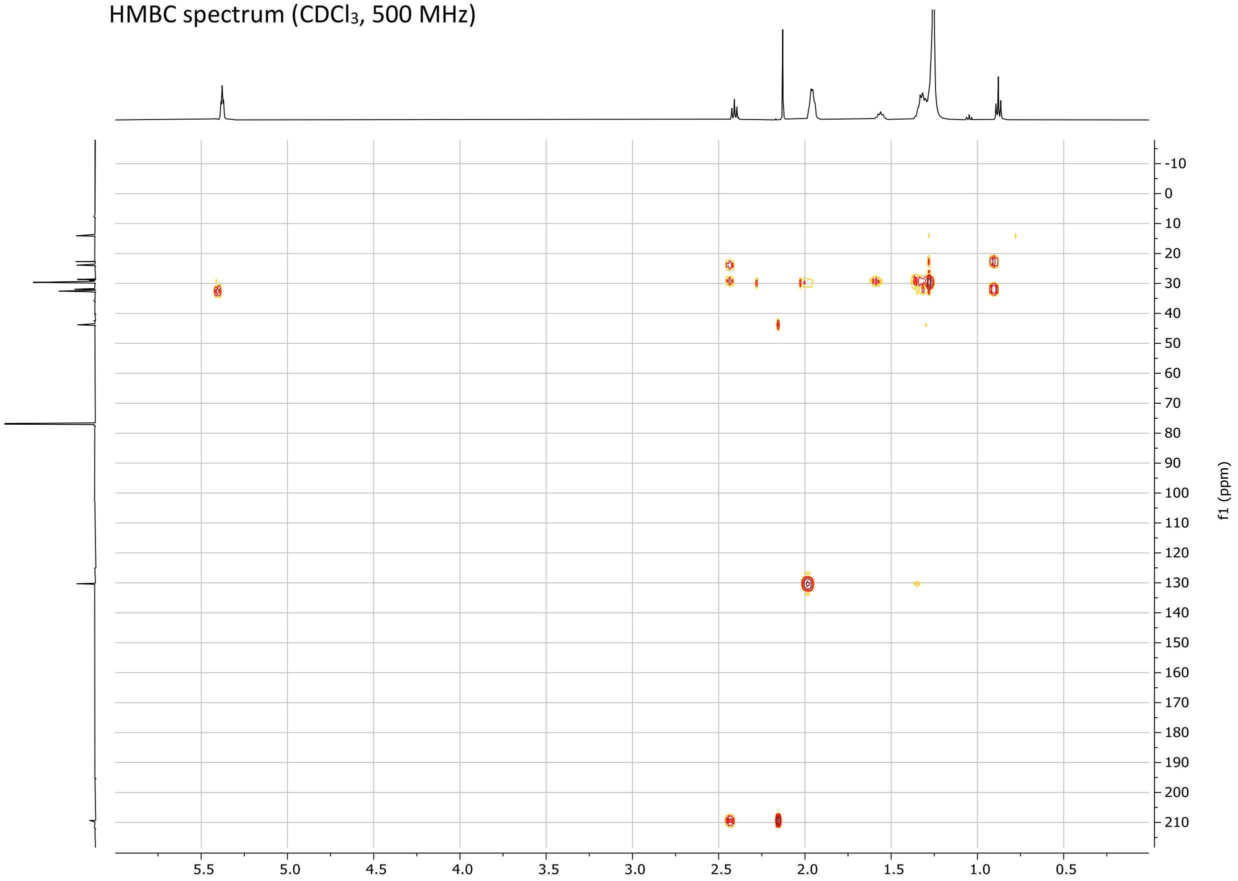

HSQC spectrum ($CDCl_3$, 500 MHz)

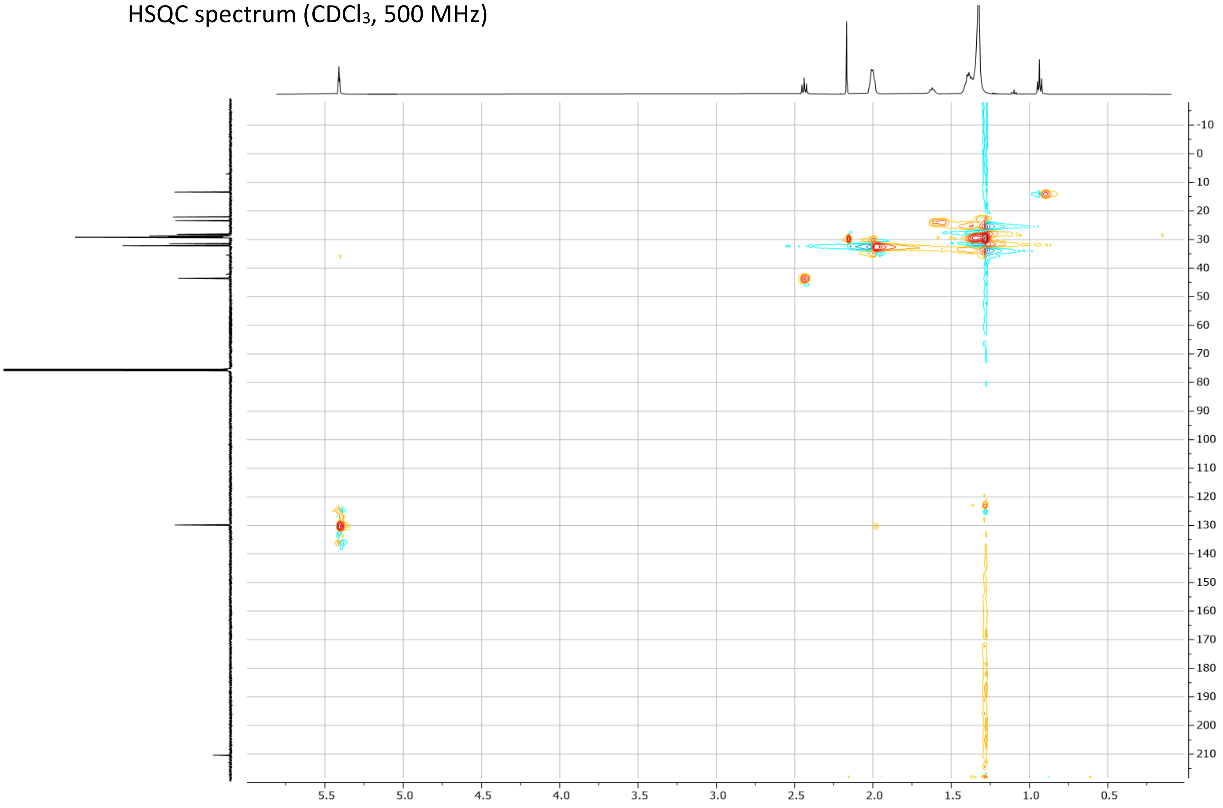

Alkenone T1 Relaxation Times Determination. T1 values for alkenone ^{1}H NMR signals were determined using a Bruker Avance inversion recovery experiments with delay times between 0.01 and 15.00 seconds. Figures S1 – S3 show the overall graph, magnifications at relevant peaks, and the curve fitting to an exponential decay.

Figure S2. Inversion Recovery Experiment on Pure Alkenones – Full Spectrum.

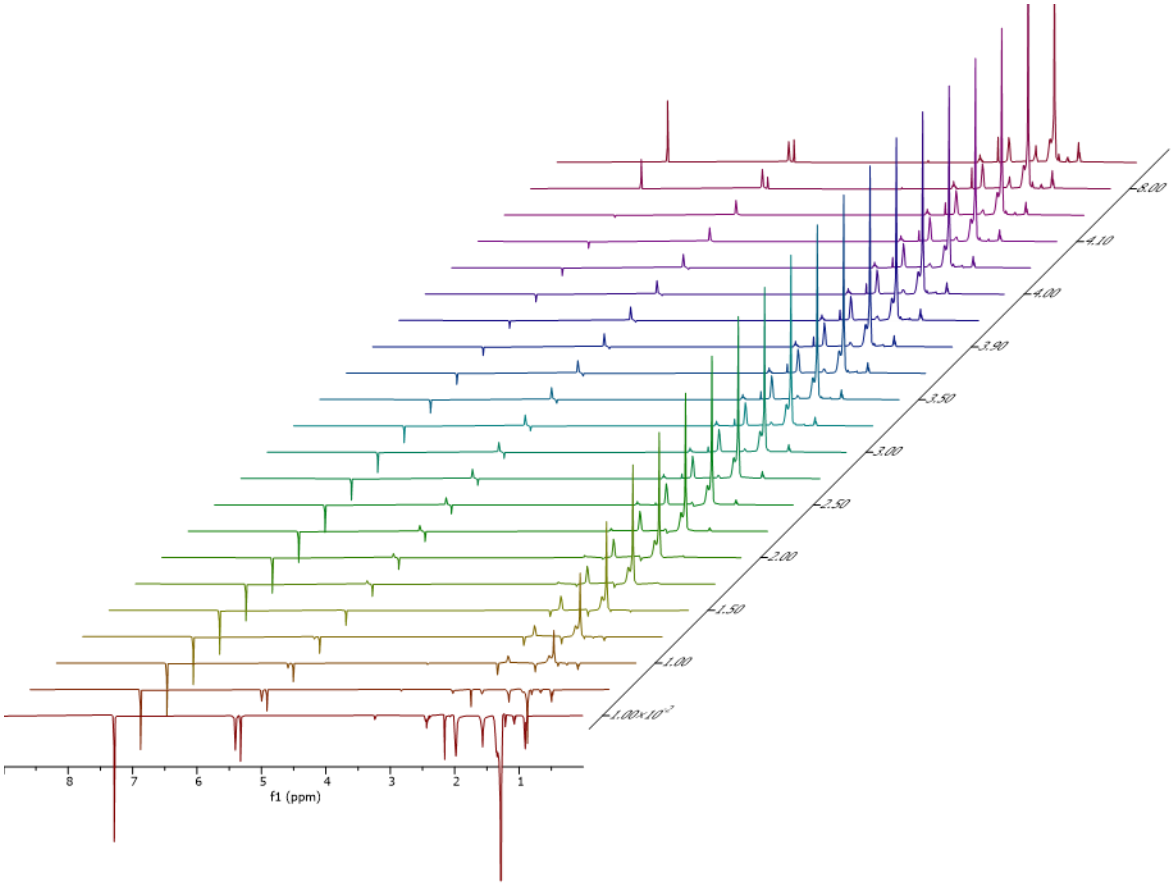

Figure S3. Expansions of inverse recovery spectra for signals belonging to methyl (2.13 ppm) and ethyl (1.05 ppm) alkenones.

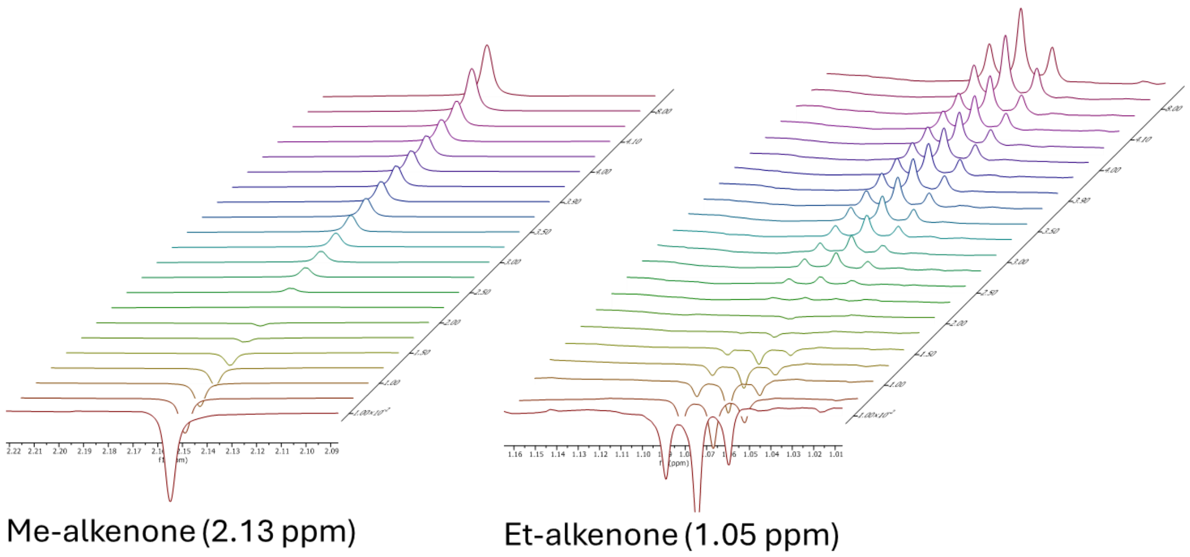

Figure S4. Integration Areas Fitted to Exponential Decay and calculation of T1.

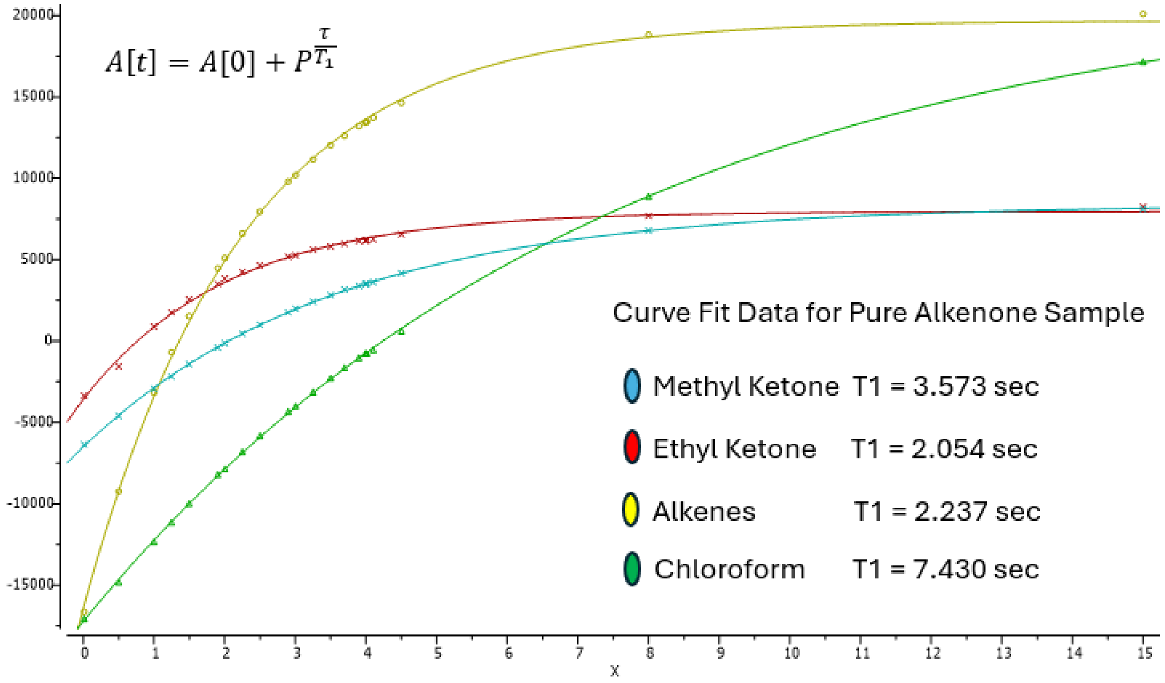

Figure S5. Mass Spectra of GC×GC Peaks A-F.

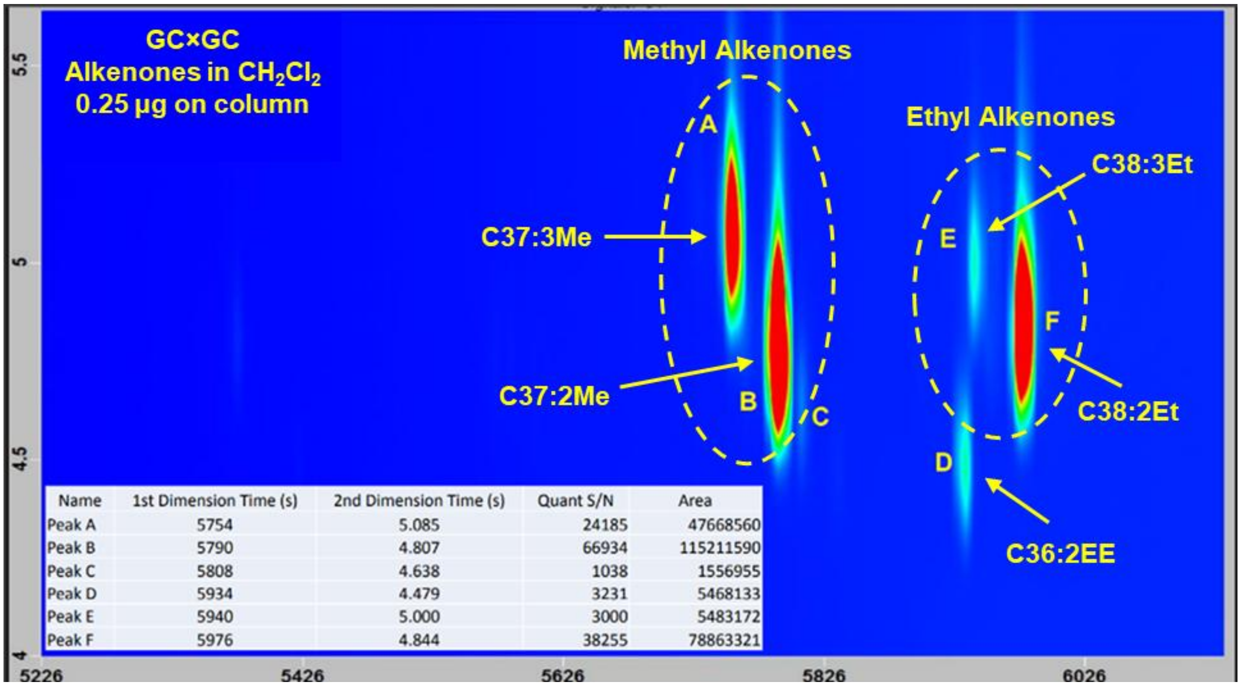

Name	1st Dimension Time (s)	2nd Dimension Time (s)	Quant S/N	Area
Peak A	5754	5.085	24185	47668560
Peak B	5790	4.807	66934	115211590
Peak C	5808	4.638	1038	1556955
Peak D	5934	4.479	3231	5468133
Peak E	5940	5.000	3000	5483172
Peak F	5976	4.844	38255	78863321

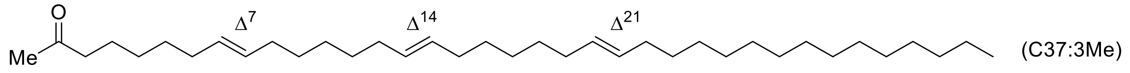

Chemical Formula: $C_{37}H_{68}O$
Exact Mass: 528.5270

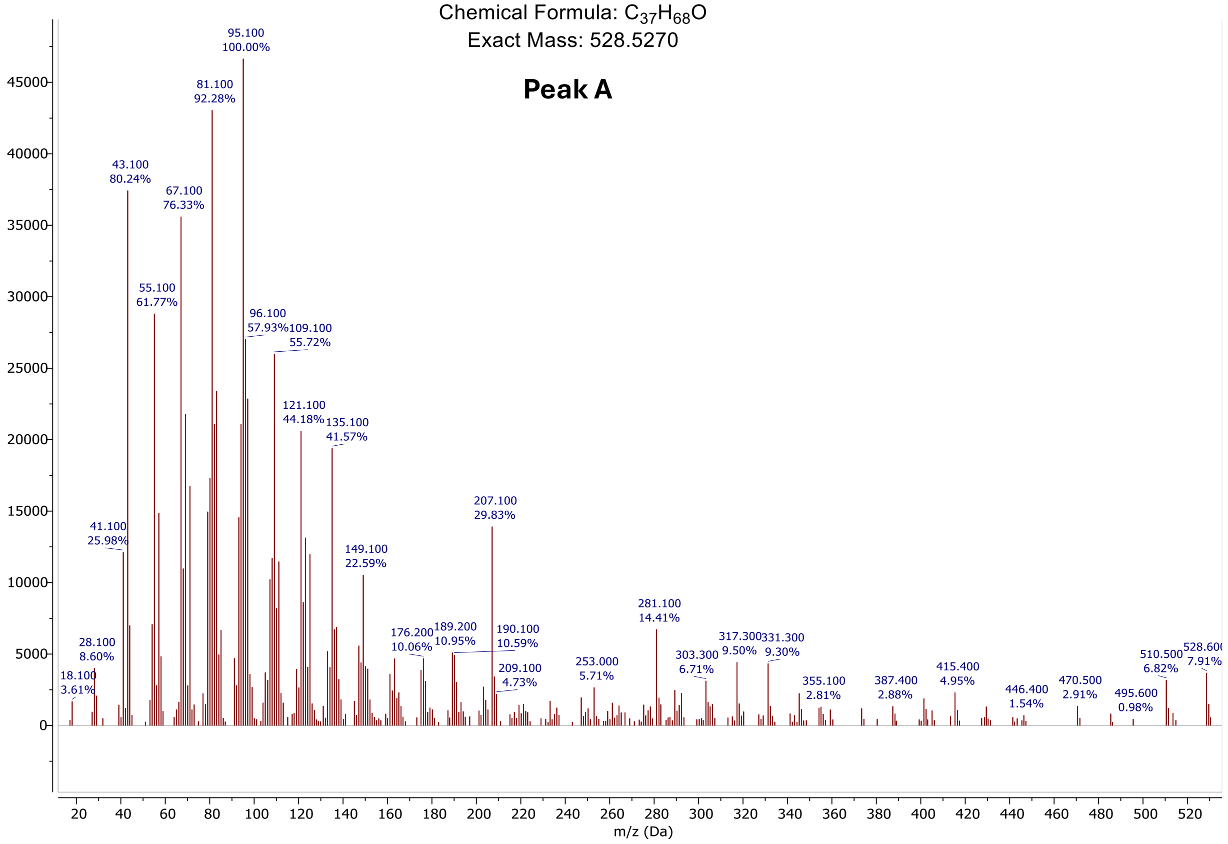

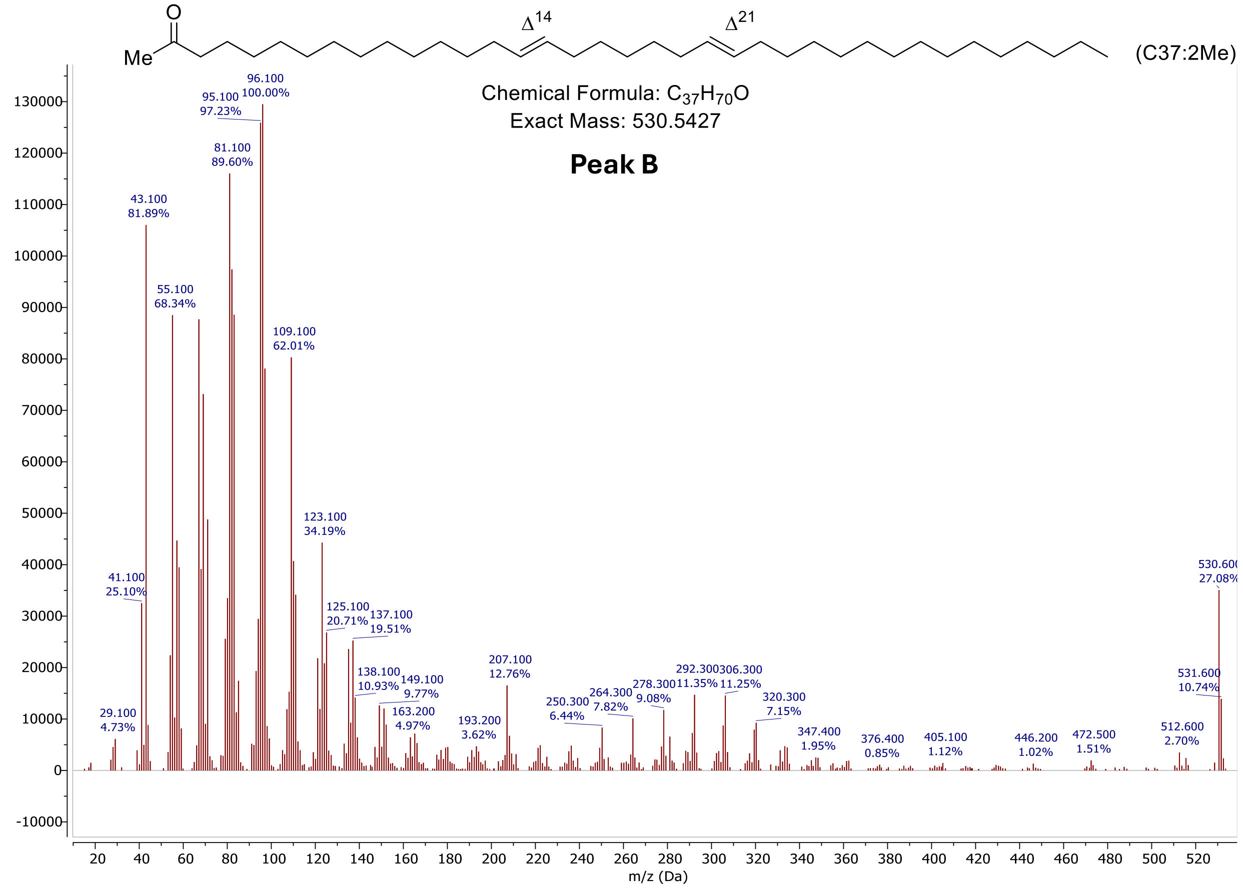
Δ14
Δ21
Me
O
(C37:2Me)
Chemical Formula: C37H70O
Exact Mass: 530.5427
Peak B
m/z (Da)

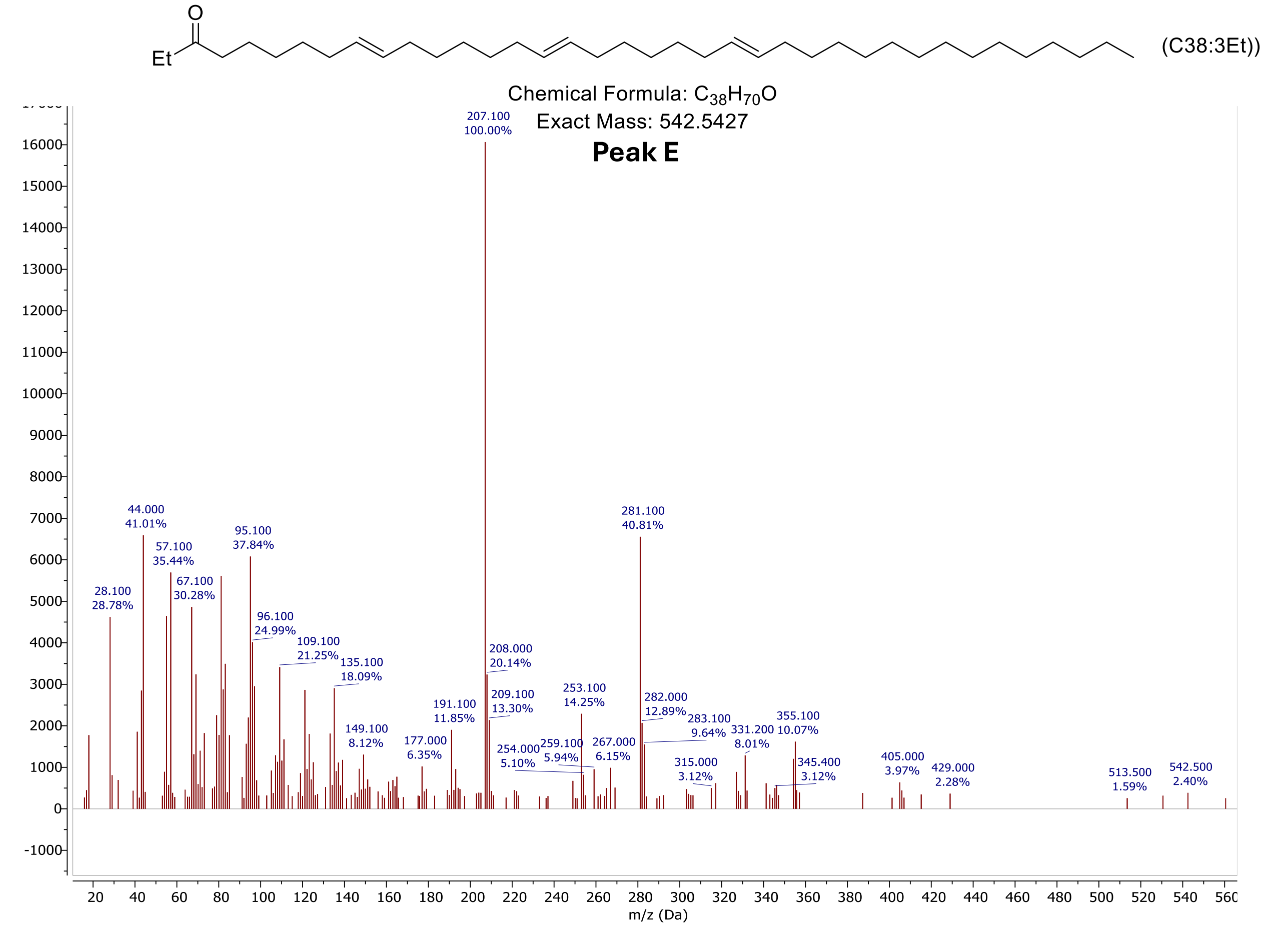
O
Et
(C38:3Et))
Chemical Formula: C38H70O
Exact Mass: 542.5427
Peak E
m/z (Da)

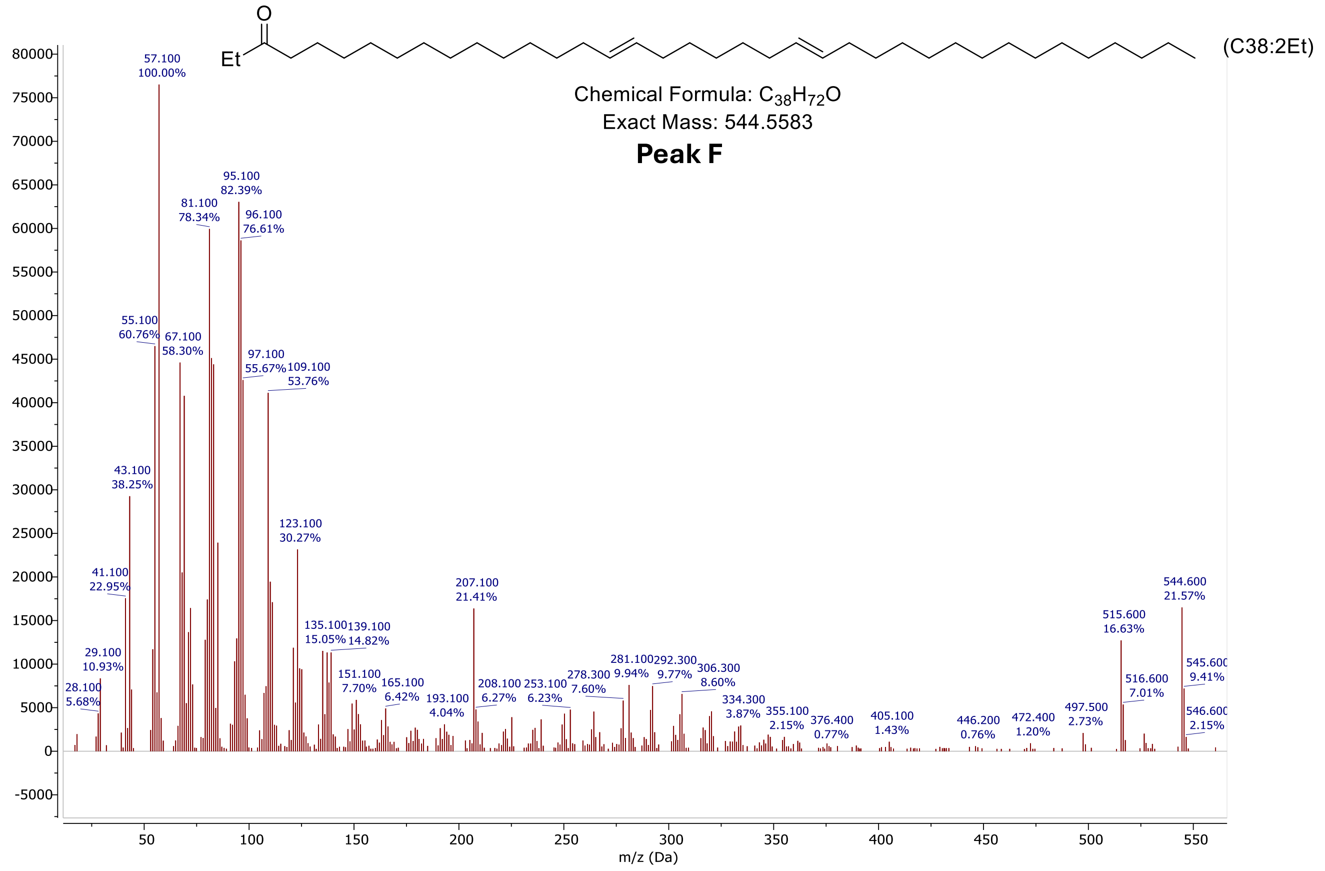

Et
O
(C38:2Et)
Chemical Formula: $C_{38}H_{72}O$
Exact Mass: 544.5583
Peak F
57.100 100.00%
95.100 82.39%
81.100 78.34%
96.100 76.61%
55.100 60.76%
67.100 58.30%
97.100 55.67%
109.100 53.76%
43.100 38.25%
123.100 30.27%
41.100 22.95%
207.100 21.41%
544.600 21.57%
515.600 16.63%
29.100 10.93%
28.100 5.68%
m/z (Da)

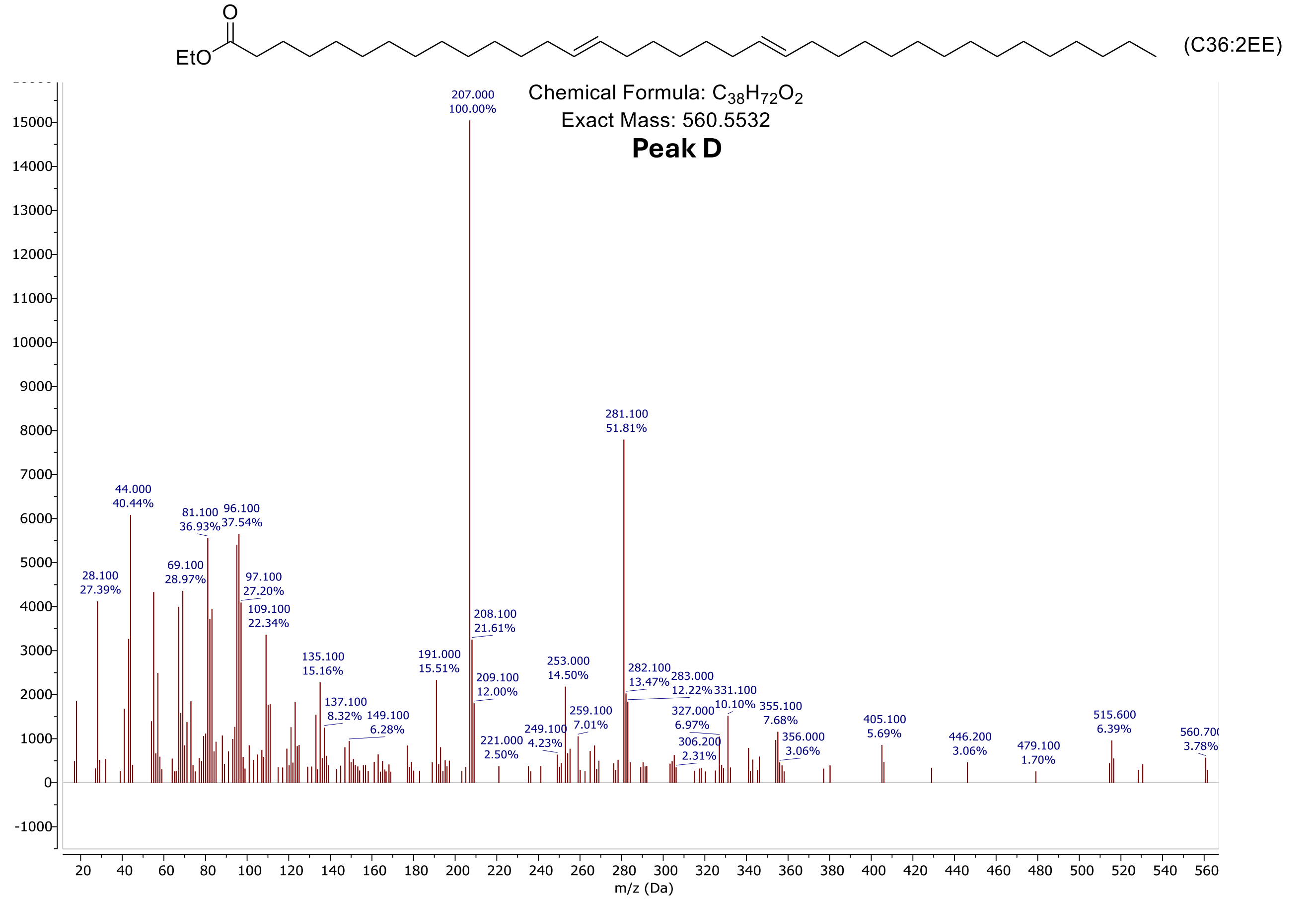

EtO
O
(C36:2EE)
Chemical Formula: $C_{38}H_{72}O_2$
Exact Mass: 560.5532
Peak D
207.000 100.00%
281.100 51.81%
44.000 40.44%
96.100 37.54%
81.100 36.93%
69.100 28.97%
28.100 27.39%
97.100 27.20%
109.100 22.34%
208.100 21.61%
515.600 6.39%
560.700 3.78%
m/z (Da)
